# Context matters in genomic data sharing: a qualitative investigation into responses from the Australian public

**DOI:** 10.1186/s12920-023-01452-8

**Published:** 2023-04-01

**Authors:** Vanessa Warren, Christine Critchley, Rebekah McWhirter, Jarrod Walshe, Dianne Nicol

**Affiliations:** 1 School of Law, University of Tasmania, Sandy Bay, TAS Australia; 2 School of Health Science, Swinburne University of Technology, Hawthorn, VIC Australia; 3 School of Medicine, Deakin University, Waurn Ponds, VIC Australia

**Keywords:** Genomic data sharing, Benefit sharing, Future use, Commercialization, Public attitudes, Governance, Genetic data

## Abstract

**Background:**

Understanding public attitudes to genomic data sharing is widely seen as key in shaping effective governance. However, empirical research in this area often fails to capture the contextual nuances of diverse sharing practices and regulatory concerns encountered in real-world genomic data sharing. This study aimed to investigate factors affecting public attitudes to data sharing through responses to diverse genomic data sharing scenarios.

**Methods:**

A set of seven empirically validated genomic data sharing scenarios reflecting a range of current practices in Australia was used in an open-ended survey of a diverse sample of the Australian public (n = 243). Qualitative responses were obtained for each of the scenarios. Respondents were each allocated one scenario and asked five questions on: whether (and why/not) they would share data; what sharing would depend on; benefits and risks of sharing; risks they were willing to accept if sharing was certain to result in benefits; and what could increase their comfort about sharing and any potential risk. A thematic analysis was used to examine responses, coded and validated by two blinded coders.

**Results:**

Participants indicated an overall high willingness to share genomic information, although this willingness varied considerably between different scenarios. A strong perception of benefits was reported as the foremost explanation for willingness to share across all scenarios. The high degree of convergence in the perception of benefits and the types of benefits identified by participants across all the scenarios suggests that the differentiation in intention to share may lie in perceptions of risk, which showed distinct patterns within and between the different scenarios. Some concerns were shared strongly across all scenarios, particularly benefit sharing, future use, and privacy.

**Conclusions:**

Qualitative responses provide insight into popular assumptions regarding existing protections, conceptions of privacy, and which trade-offs are generally acceptable. Our results indicate that public attitudes and concerns are heterogeneous and influenced by the context in which sharing takes place. The convergence of key themes such as benefits and future uses point to core concerns that must be centred in regulatory responses to genomic data sharing.

**Supplementary Information:**

The online version contains supplementary material available at 10.1186/s12920-023-01452-8.

## Background

Significant advances have been made in human genomics research over the past few decades, albeit with a slower translation into tangible benefits in clinical practice. Real improvements in clinical care will only arise if there is better understanding of the influences of genomics on health, whether at the level of the individual, the local community, or globally [1]. More work is therefore needed to better understand the consequences of genome variation on health both within and across populations. Progress relies on sharing of genomic data between individuals, research laboratories and clinics globally. There are many reasons why individuals may be either enthusiastic or concerned about having their genomic data shared [2].

Results from the global Your DNA, Your Say survey are already providing a useful account of how public views on the donation of genomic data can vary between countries, and between different cohorts within countries [3]. Other findings published to date provide an indication of the types of factors that might influence public attitudes towards genomic data sharing (GDS), and how these can vary between jurisdictions. Across the board, individuals tend to have highest levels of trust in their own doctors and lowest in researchers working in private companies [4]. Between cohorts, it appears that people who consider genomic information to have special significance relative to other forms of health information (so-called genetic exceptionalists) may be more willing to donate genomic data than the rest of the population [5].

There is a large and growing body of other public opinion research on the attitudes of particular cohorts of individuals towards genomic data sharing. These cohorts include research participants [6], patients and their families [7, 8], particular groups of individuals within society (including Indigenous peoples [9, 10] and other marginalised communities and cultures [11, 12]) and members of the broader public [13, 14]. A comprehensive review by Shabani et al. provides further detail on studies published before 2014 on research participant and public attitudes towards genomic data sharing [15].

There are a wide variety of locations where genomic data may be generated and used, ranging from the hospital clinic, to the university’s research laboratory, to the technology company’s laboratory and beyond. There is a body of public opinion research indicating that the context within which genomic data is generated and shared is likely to influence an individual’s attitude towards sharing [13]. In Australia for example, we already know that intention to participate in GDS changes if private companies are involved, compared with sharing within and between public research and clinical laboratories [16, 17].

Recognising the value of this extensive body of public opinion research, in formulating policy responses to GDS there is nevertheless an important gap in the literature. There is a lack of research exploring how the factors influencing public attitudes towards GDS might vary depending on the circumstances within which genomic data is generated and shared. Does it matter, for example, if genomic data is collected for clinical purposes, or for research purposes, or for a clinical trial, or for direct-to-consumer genetic testing? Does the way in which the data is used matter? Research exploring these questions has the capacity to play an important role in policy development and regulatory reform. Regulation of the collection, use and sharing of genomic data currently remains very much siloed along traditional lines. There are important regulatory distinctions in relation to: consent and non-consent; samples and data; deidentified and identified data; clinical and research data; public and private organisations [18]. These regulatory distinctions remain, even though in practice the hard borders between each of these categories are dissolving. An understanding of differences in public attitudes in different data sharing scenarios will assist in guiding the much needed reform process.

The research reported in this article focuses specifically on a preliminary investigation into how broad public views on GDS can vary within a single country (Australia) depending on the context within which the genomic data is generated and shared. This research is part of a larger project exploring how Australia can make best and most responsible use of the vast amount of genomic data being generated globally, for the benefit of our communities, science, healthcare and the economy. We recognise that achieving this aim requires strategies to ensure fundamental human rights, public trust and freedom of research are protected and innovation is facilitated. The overarching aim of this project is to provide best-practice guidance for the design of regulatory and governance strategies to achieve these ends in Australia. The project includes the creation and use of a series of GDS scenarios to map legal and quasi-legal facilitators and barriers to sharing, and to assess their roles in promoting public trust, using evidence-based processes and law reform methodology [19].

The aspect of the project reported in this article involved the use of simplified versions of the GDS scenarios in semi-structured questionnaires. The aim was to determine if and/or how public attitudes towards GDS may be influenced by the context in which the genomic data is generated and shared.

## Method

### Participants

An online semi-structured questionnaire was designed to obtain qualitative reactions to seven scenarios. Respondents were recruited by Qualtrics [20], a multinational survey and analytics company, over the period 12–20 February 2020. Qualtrics outsources recruitment to various companies that provide online panel members who have consented to participate in surveys for a small incentive, typically points that can be redeemed for products or services. Since companies use different methods to promote survey completion response rates are difficult to obtain. Recruitment employed a quota system to ensure a diversity of views were captured, using categories for stratification that were either previously found or could reasonably be expected to influence attitudes to genomic data sharing in the scenarios provided [21]. All participation was anonymous.

### Data collection

A survey instrument was constructed by the research team to obtain reactions to seven externally validated prototypical scenarios that categorize current Australian GDS practices [19]. The scenarios ranged from the sharing of genomic data in clinical, research, biobank, and data repository settings, through to citizens sharing information generated through a direct-to-consumer company. Some previous studies have employed hypothetical vignettes in exploring public attitudes to GDS [21–23]; it is anticipated that the use of detailed, validated scenarios explicitly grounded in real-world data sharing practices will provide methodological rigour and strategic relevance to regulatory recommendations [19]. While these scenarios do not represent all current and emergent contexts in which GDS takes place, external validation confirmed that they captured a meaningful cross-section of prototypical genomic data sharing settings in Australia at the time of development [19]. The scenarios included a range of variables reflecting potential ethical, legal and social challenges identified through extensive qualitative interviews with stakeholders engaged with GDS in these contexts. These variables included the data source, provider, intermediary and user, the purpose of sharing, type of data, storage and data security, return of results, consent procedures and monitoring and oversight.

McWhirter et al. originally developed six scenarios [19], which were subsequently adapted for this component of the project to ensure they were accessible to a lay public with an average education level of Year 10. One scenario depicting GDS in a clinical setting for diagnostic purposes was repeated to compare an adult to infant patient to gauge reactions associated with making decisions on behalf of a minor. A summary of the seven scenarios (S1-S7) used in this study is provided in Table 1. The full description of each scenario as read by participants can be found in supplemental materials (see Additional file 1).

**Table 1:** Summary descriptions of the prototypical genomic data sharing scenarios

Scenario number	Scenario Short In-Text Title	Scenario summary	Number of participants
S1	Diagnosis of infant	Clinician-led sharing of de-identified genomic and medical (phenotype) infant patient records for diagnosis of a rare condition with trusted doctors face to face and via a hospital computer system.	40
S2	Diagnosis of adult	Clinician-led sharing of de-identified genomic and medical (phenotype) adult patient records for diagnosis of a rare condition with trusted doctors face to face and via a hospital computer system.	30
S3	Association study	Clinician-led sharing of de-identified genomic, leftover tissue from a surgical procedure and medical records to an international consortium led genome wide association study approved by a university ethics committee. Data can only be accessed and analysed by a cloud-based platform. General though not individual research results are shared with donors via regular newsletters.	41
S4	Waived consent	A pancreatic cancer researcher who has sequenced the genomes from tissue obtained from an Australian tissue bank comprising samples from Aboriginal and Torres Strait Islander participants needs to submit the de-identified results to an international data repository for publication purposes via a cloud-based platform. A consent waiver is obtained from a university ethics committee, as consent has only been obtained for further use in pancreatic cancer research. Individual results cannot be returned despite international third-party researchers, approved by a data access committee, detecting preventable risk.	30
S5	Private clinical trial	A privately funded clinical trial to develop a new treatment genotypes blood samples (linked to medical records) obtained from a privately controlled biobank. Donors are able to withdraw from the trial, but not from the biobank and future use as their sample is de-identified.	32
S6	Biobank	A data access committee approved team of Australian researchers access de-identified genome sequences from four international biobanks. A data transfer agreement stipulates that the data can only be used for specific purposes that are within the scope of the original consent provided, however most have provided broad consent. Re-identification of data is made difficult by the biobank’s use of data encryption, meaning participants cannot be contacted and will not know how their data will be used.	32
S7	Direct-to-consumer	A direct-to-consumer genetic testing customer uploads her genetic health risk report online to investigate a diagnosis and seek similar others. Her identity is protected by a private messaging system but is linked to actual email addresses, allowing private contact. Her data is used by the company for unknown research purposes and without acknowledgement of intellectual property rights.	40

Where possible, language was simplified to be accessible to a lay audience, including referring to genomic data as ‘genomic information’. In addition, each scenario included definitions of concepts (e.g., accredited, whole genome sequence, medical information), entities (e.g. university ethics committee, consortium, data access committee), and systems (e.g., cloud based platform, clinical trial, data transfer agreement) that we anticipated could be unfamiliar. The terms were bolded, with participants being instructed to “hover your mouse or cursor over the highlighted word in the scenario” to obtain a definition.

Five open-ended questions were developed to capture respondents’ views on whether or not respondents would share their genomic data in the given scenario, why they would or would not be happy to share, what their decision to share would depend on, the perceived benefits and risks of sharing, which risks they would be willing to accept if sharing was certain to result in benefits, and views on what would increase comfort about sharing and any potential risks associated with sharing genomic information. This approach was employed to explore if, and/or how, participant responses varied between different sharing scenarios, enabling the identification of themes for further investigation with a view to developing empirically supported recommendations for regulatory reform in Australia. Participants had an unlimited character count within the platform default of 20,000 characters in which to give their responses to open-ended questions. The whole survey instrument is provided in supplemental materials (see Additional file 2).

Before respondents received the scenario and questions, they were instructed to view a two-minute video to ensure familiarity with concepts such as genomes, genes and sequences. To check that respondents were familiar with the key aspects covered in the video, six true or false questions were asked (see Additional file 2). A between groups design was employed, where respondents were randomly presented with one of the seven scenarios. An extensive quota system was used to obtain a range of views rather than statistical representation of the Australian population. Quotas were designed to recruit a minimum of 1 male and 1 female respondent per scenario in each of the following categories: age (< 50 years, ≥ 50 years), education (university educated, not university educated), indigeneity (Aboriginal and/or Torres Strait Islander, not Aboriginal and/or Torres Strait Islander), country of birth (Australian born, not Australian born), location (urban, rural or remote), parental status (have children, do not have children), experience of a diagnosis of a serious health condition personally (yes, no) or with an immediate family member (yes, no).

With one exception, the quota was achieved for all scenarios and demographic characteristics. The exception was that Scenario 2 and Scenario 5 were not viewed by an Aboriginal and/or Torres Strait Islander respondent (see Additional file 3). Although the number of respondents receiving each scenario was not equal (range = 30–41), chi-square analyses revealed that there were no significant (at *p* < 0.05) differences between the scenario received and all demographic variables (see Table 2).

**Table 2:** Pearson chi-square tests for scenario assignment by quota demographics

Variable	χ2	df	*p*
Age group	4.92	6	0.554
Education	3.17	6	0.788
Disease—Personal	7.24	6	0.300
Disease—Family	9.45	6	0.150
Cultural background	9.07	6	0.170
Australian born	6.02	6	0.421

Due to insufficient sample size, the test could not be performed for the Aboriginal and/or Torres Strait Islander variable

### Data analysis

In seeking to identify themes and trends in participant responses to the different scenarios as the starting point for further investigation, the analysis of this survey reflects a broadly post-positivist orientation. A thematic analysis approach [24] was used to examine the responses to the five questions initially across all scenarios and then within each. Initially two independent coders, blinded to scenarios, read all responses to extract meaning or themes from their content and sentence structure. Themes and their meaning were then compared by the two coders, which resulted in additional themes being created and some being merged or omitted. This coding structure was then presented to all authors for validation and comment, which resulted in no changes. The final structure was then used by both original coders to recode all comments independently. Disagreements were found for 42 responses (4.03%) and all were resolved by discussion between the coders. Finally, themes were examined across the seven scenarios. The final coding scheme with definitions and example quotes for each question can be found in supplemental materials (see Additional file 4). SPSS Version 26 was used to conduct descriptive statistics and logistic regression results. The latter involved the scenario received predicting the likelihood of sharing (yes/no/depends), perceived benefits (yes/no) and risks (yes/no).

## Results

### Participants

A total of 243 members of the public took part in this survey. All but one quota was achieved, facilitating the inclusion of a range of views and attitudes (see Additional file 5). All were Australian residing in all states and territories except the Northern Territory (NSW = 61, VIC = 71, QLD = 55, WA = 26, SA = 21, TAS = 6, ACT = 3). Approximately half were female (49.2%) and under the age of 50 years (46.5%), with 30% indicating that they had either an undergraduate or postgraduate university qualification. Most were Australian born (76.1%) and lived in an urban area (81.9%), with 2.5% identifying as Aboriginal and/or Torres Strait Islander. Over half had children (61.3%), and a minority had experienced a diagnosis of a serious health condition personally (25.9%) or via an immediate family member (39.9%).

### Viewing time for video

The time between clicking on the video link to moving to the next page of the survey suggested that five respondents did not watch it in its entirety (i.e. seconds were < 120; range = 81.33–105.88). The mean viewing time was 203.08 s (SD = 174.71; range = 81.33–1612.05 after removing 3 extreme outliers). The majority of respondents answered all six questions correctly (74.9%), and the mean number of questions correct was 5.68 (SD = 0.65; range = 1–6).

### Responses

***Question 1: If you were the (patient/parent/research participant) would you be happy for the (doctor/researcher) to share the results?***

Respondents reported a high intention to share their genomic data, with an overall mean of 71.2% across all the scenarios (Table 3). Within this result however, intention to share varied dramatically between scenarios, with the highest intention to share at 90.0% in Scenario 2 (diagnosis of an adult) and the lowest at only 30.0% in Scenario 7 (direct-to-consumer). The highest intention to share, found among the three clinical scenarios (S1, S2 and S3) was closely followed by the biobank and repository scenarios (S4 and S6), which were not significantly different from the three clinical scenarios in terms of intending to share versus not intending or selecting depends. Intention to share was significantly lower for both the direct-to-consumer (S7) and privately run clinical trial (S5) scenarios compared to all others, with one exception. That is, the tendency to respond ‘yes’ to sharing compared to ‘no’ for S5 was not significantly different from the researcher-led repository (S4). The privately run clinical trial (S5) was not significantly different from S7 in terms of tendency to share (or not), but the likelihood of answering “depends” (relative to “yes”) was significantly higher for the direct-to-consumer scenario (S7) compared to the private clinical trial (S5).

**Table 3:** Percentage of responses to sharing, perceived benefits and risks across scenario

	Scenario	If you were the patient/parent of the patient/research participant in this scenario, would you be happy for the doctor/researcher to share your/their results?			If you did decide to share your/your child’s genomic information in this situation, do you think there would be any benefits or positive consequences?		If you did decide to share your [your child’s] genomic information in this situation do you think that there would be any risks or negative consequences?		
		Yes	No	Depends	Yes	No	Yes	No	n
S1	Clinician-led sharing of clinical genomic data for diagnosis (infant patient)	82.5	2.5^Y5,Y7^	15.0^Y7^	92.5	7.5^Y5^	30.0	70.0	40
S2	Clinician-led sharing of clinical genomic data for diagnosis (adult patient)	90.0	3.3^Y5,Y7^	6.7^Y7^	96.7	3.3^Y5^	30.0	70.0	30
S3	Clinician researcher-led sharing of genomic data for genome wide association study	87.8	4.9^Y5,Y7^	7.3^Y7^	82.9	17.1	31.7	68.3	41
S4	Researcher-led sharing of pre-existing genomic data for research based on waiver of consent, indigenous findings and return of results	73.3	6.7^Y7^	20.0^Y7^	90.0	10.0	26.7	73.3	30
S5	Sharing of genomic data obtained by a company-sponsored clinical trial based on participant consent	62.5	21.9	15.6^Y7^	71.9	28.1	46.9	53.1	32
S6	Researcher-led sharing of genomic data for research from multiple sources	76.7	10.0^Y7^	13.3^Y7^	86.7	13.3	23.3	76.7^Y7^	30
S7	Citizen-led sharing of genetic data from direct-to-consumer testing	30.0	30.0	40.0	90.0	10.0	47.5	52.5	40
Total		71.2	11.5	17.3	87.2	12.8	34.2	65.8	243

Superscripts denote significant differences between a scenario and another scenario. ^Y(number)^indicates a significant difference (at least at *p* < 0.05) between the category relative to a Yes response across scenarios

Findings from the thematic analysis are characterised by a largely homogeneous perception of benefits and positive consequences, and a heterogeneous response to perceived risk and negative consequences across the different scenarios.

**If yes, why would you be happy to share your genomic information in this scenario?**

**Fig. 1:**
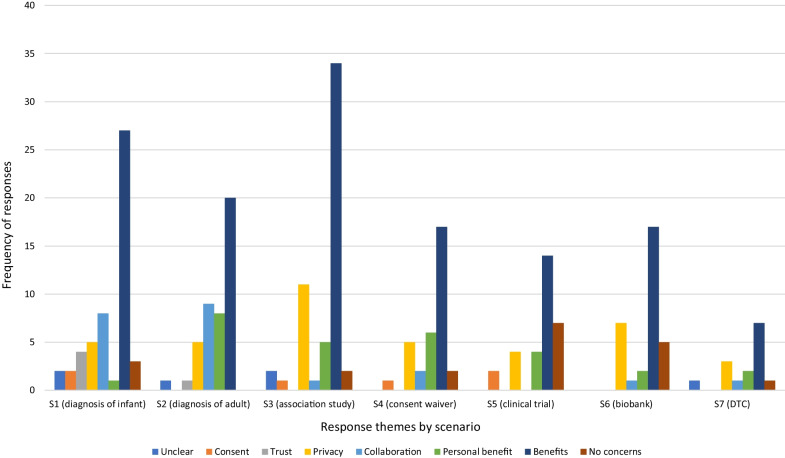
Distribution of themes for responses to *Q1. Would be happy to share* by scenario

The expectation of benefits was the dominant explanation for willingness to share data across all the scenarios (Fig. 1). Although some respondents, particularly in the two clinical diagnostic scenarios (S1 and S2), linked sharing to personal benefit, for example, “*to ensure that I get the best care*” (Younger Woman, S2), most framed benefit in terms of helping others, and in the researcher-led scenarios particularly, scientific advancement “*to help other people with the same issues its really great*” (Younger Man, S6), “*I am all for furthering the advancement of research*” (Older Woman, S2).

Others indicated that their willingness to share was based on a belief that “*…the possible benefit of sharing the information would outweigh the risks in my estimation*” (Older Woman, S2), on the basis of their trust in medical professionals and data security in the diagnostic scenarios (S1 and S2) “*I would like to think that a doctor has my (or others) best interests at heart*” (Younger Man, S2), and the understanding that data would not be shared without their consent “*I wouldn’t have agreed to the study in the first place if I were not happy with the terms*” (Younger Woman, S5).

**If no, why would you not be happy to share your genomic information in this scenario?**

**Fig. 2:**
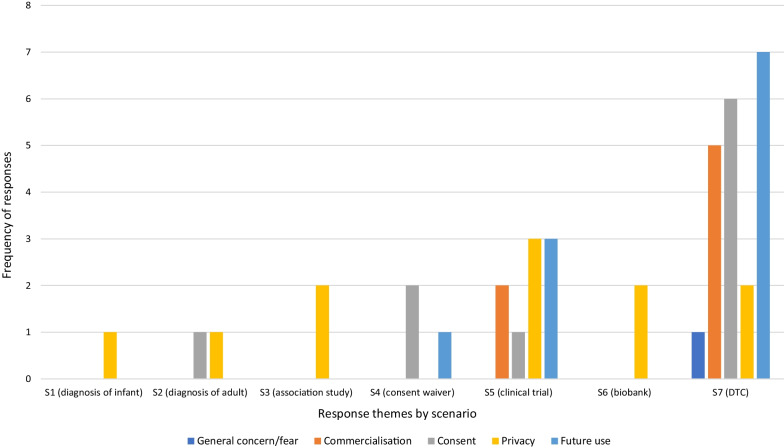
Distribution of themes for responses to Q1. *Would not be happy to share* by scenario

Privacy was an important explanation for not sharing data, mentioned in all scenarios except S4 (waiver of consent) for this question (Fig. 2). Participants expressed a desire to “*…keep my details private*” (Younger Woman, S3), along with concerns about re-identification and data security, particularly in the private clinical trial (S5) and biobank (S6) scenarios.

Concerns about future use were also important to respondents, particularly in the direct-to-consumer scenario (S7), with respondents unwilling to share data “*without further clarification on how the information will be shared into the future*”(Younger Woman, S3).

In a pattern that follows throughout the other questions, commercialisation was a recurrent theme among respondents in the two scenarios involving private companies (S5 and S7) with respondents offering explanations such as “*I don’t believe that my DNA should be used for commercial purposes that I am unaware of*” (Younger Person, S7), and “*its my information. What is stopping the company from selling that information*” (Younger Man, S5).

**If depends, what would sharing depend on?**

**Fig. 3:**
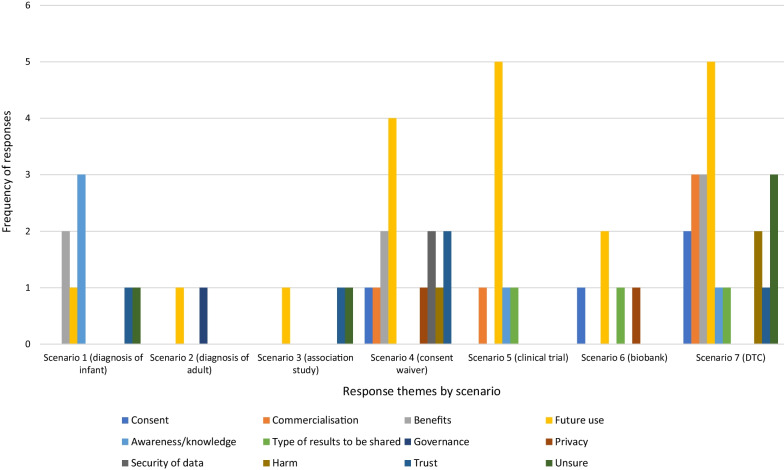
Distribution of themes for responses to Q1. *Sharing would depend* by scenario

For some respondents, the willingness to share genomic information was dependent on certain criteria being met or concerns being addressed (Fig. 3). While future use was a factor in determining willingness to share across all scenarios, it was particularly prevalent in the two associated with private industry (S5 and S7) and the researcher-led project with a consent waiver (S4). Respondents indicated that agreeing to share “*…depends on who it is being shared with, their reason for needing it and what they will actually do with the outcome*” (Older Man, S4), while others highlighted concerns with further sharing outside the proposed scenario “*how far does this information go? Or is it possible that company may share it further*”? (Older Woman, S7).

Other themes appearing in small numbers include concerns about commercialisation in S4 (waiver of consent), S5 (private clinical trial) and S7 (direct-to-consumer); the impact and urgency of benefits in S1 (diagnosis of infant), S4 (waiver of consent) and S7 (direct-to-consumer); and the need for explicit consent in S4 (waiver of consent), S6 (biobank) and S7 (direct-to-consumer).

***Question 2: If your genomic information was shared in this situation, do you think there would be any benefits or positive consequences?***

Respondents saw all scenarios as likely to result in benefits regardless of intention to share (see Table 3); 90.0% of respondents in Scenario 7 (DTC) reported perceived benefits for sharing in their scenario, for example, despite only 30.0% of these respondents indicating an outright intention to share their genomic data. However, significantly more respondents indicated that benefits would result from the two clinical diagnostic scenarios (S1 and 2) compared to the privately run clinical trial scenario (S5).

**What do you think those benefits or positive consequences could be?**

**Fig. 4:**
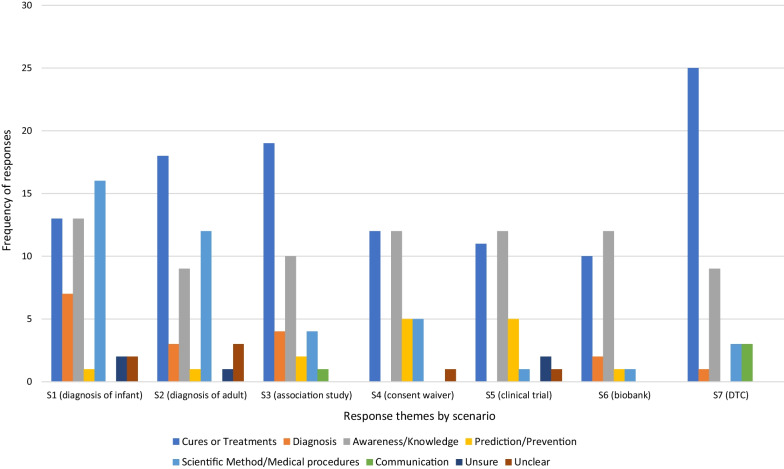
Distribution of themes for responses to Q2. *There would be benefits or positive consequences* by scenario

Among those who believed there would be benefits or positive consequences to sharing their genomic information the strongest theme (Fig. 4) was an anticipation of cures and treatments, which participants linked to personal benefits, general benefits, and in many cases both “*it would definitely help to improve my health now and into the future. It also helps to identify those at risk to certain conditions and enables labs to improve treatments for those issues*” (Older Woman, S5).

Many respondents across the scenarios showed a clear expectation that sharing data would have benefits for knowledge “*I expect they would be able to learn new information or confirm previously gathered information*” (Younger Woman, S5), and benefits to scientific methods and/or medical procedures*,* through “*better use of time and resources to target the condition*” (Younger Man, S2). This theme appeared in all scenarios despite the different purposes and methods of sharing genomic data described in each scenario, and was particularly strong in the clinical diagnosis scenarios (S1 and 2).

**Why do you think that there would be no benefits or positive consequences?**

**Fig. 5:**
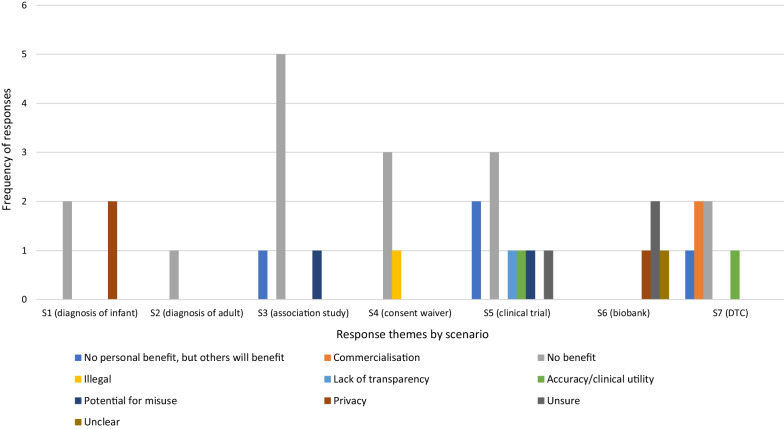
Distribution of themes for responses to *Q2. There would not be benefits or positive consequences* by scenario

Each scenario included respondents who answered that they thought there would not be any benefits or positive consequences to sharing their genomic information, with S5 (private clinical trial) returning the greatest number of ‘no’ responses (Fig. 5). Though responses were few and somewhat fragmented thematically for this question, they generally fell under a scepticism of the benefits of sharing their genomic information or concerns regarding specific practices and potential consequences. In the former, respondents explained that they couldn’t imagine any benefits personally “*I don’t have any health problems, nor do my family so what benefit would there be?”* (Older Man, S5), or more broadly “*what’s the point? Why are they studying random DNA?*” (Younger Man, S4).

Other responses indicated concerns about privacy “*I don’t want anything shared that could identify me…*” (Younger Woman, S7), lack of transparency concealing undesirable motivations or outcomes “*I just think if someone can’t disclose what this would be used for then there must be something not too good behind it, maybe not but this info is pretty important*” (Younger Woman, S5), and suspicion of commercialisation, including potential implications for health insurance *“not for me there wouldn’t be but the person who is selling it is who would benefit by making money” (Younger Woman S7),* “*it might not be beneficial to me as I might be charged higher private healthcare in the future*” (Younger Man, S7). However, each of these themes were reported less frequently than the general theme of no benefit, which was the only response theme to appear in all scenarios.

***Question 3a: If your genomic information was shared in this situation, do you think there would be any risks or negative consequences?***

While respondents in each of the scenarios perceived risks or negative consequences they appeared far more frequently in the scenarios involving private industry (S5 and S7), although these differences were not significant. In these two scenarios close to half of the respondents (46.90% and 47.50% respectively) answered that they thought there would be risks or negative consequences to sharing their genomic data; in all the other scenarios a far greater proportion of respondents said that they perceived no risks or negative consequences, with a significant difference observed between the scenario with the fewest ‘yes’ responses, S6 (biobank), compared to S7 (direct-to-consumer) (see Table 3).

**Why do you think that there would be no risks or negative consequences?**

Of those respondents who answered ‘no’ to this question, the dominant explanation was simply that they perceived no or negligible risks in their scenario “*I cannot see any situation where there would be risks or consequences. The whole scenario looks positive to me*.” (Older Woman, S4). A smaller subset of responses in this theme linked their interpretation of negligible risk to their understanding of the nature of the information being shared “*genetic information doesn’t currently have the same risks as other forms of identification, such as bank details*” (Younger Man, S4), or to the likelihood of risks manifesting “*although it is possible to re-identify someone if you have their whole genetic sequence/information, it is far too time consuming and tedious to do so. I assume most people simply would not bother to go to that effort to do so*.” (Younger Woman, S2).

**Fig. 6:**
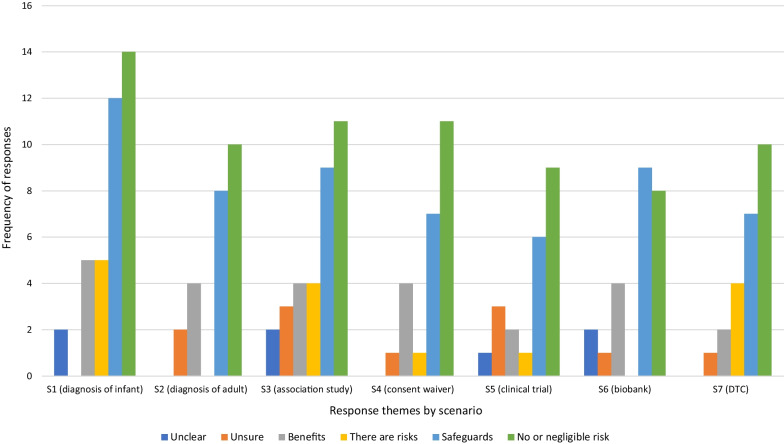
Distribution of themes for responses to *Q3. There would not be risks or negative consequences* by scenario

The no or negligible risks theme was the most frequent response in all scenarios except S6 (biobank), in which responses around safeguards were returned more frequently (Fig. 6). The safeguards theme included a number of sub-categories, primarily confidence in de-identification protocols “*I don’t see any personal risks, there are no personal identifiers*” (Younger Woman, S5), but also including trust in the professionals involved in data sharing, confidence in existing regulatory safeguards, data security, and confidentiality.

**What do you think those risks or negative consequences could be?**

**Fig. 7:**
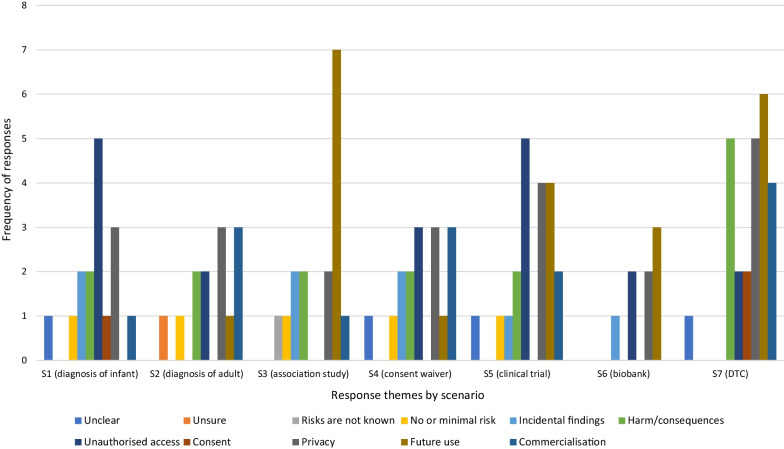
Distribution of themes for responses to *Q3. There would be risks or negative consequences* by scenario

Privacy and future use were the most frequently identified areas of risk or negative consequences across the scenarios (Fig. 7). Respondents reported concerns about future use in all scenarios except S1 (diagnosis of infant), and it was the most common theme in S6 (biobank), S7 (direct-to-consumer), and particularly S3 (association study). Risks associated with future use included sharing with unknown actors “*…you never know who’s [sic] hands it could end up in*” (Younger Man, S7), and for reasons outside the original purpose, which could lead to misuse “*genetic information could be inappropriately released for nefarious purposes*” (Older Man, S5).

Privacy, which appeared in all scenarios, included concern both that “*there is the chance of being identified…”* (Younger Woman, S3) and that specific harms to the individual may result from being identified, or identifiable, such as “*negative impact on employment, insurance, medical decisions *etc*.*” (Older Man, S4).

Concern about unauthorised access was strongest in S1 (diagnosis of infant) and S5 (private clinical trial). Responses in this theme described the possibility that data may end up in the hands of unauthorised, unscrupulous or inappropriate actors “*I suppose these is always the risk that it gets mixed up with someone else’s DNA or it gets into the wrong the hands. It would need to be secure and only shared with trusted doctors and clearly labelled*.” (Younger Man, S1). This was also associated with the possibility of hacking or data theft and, in direct contrast to respondents in the preceding section, the belief that technological safeguards are not robust enough to mitigate these risks “*online databases can be reasonably easily hacked, as such my information could be stolen and shared without my consent*.” (Younger Person, S7).

Other minor themes include commercialisation (equal first in S2 (diagnosis of adult) and S4 (waiver of consent), the anticipation of societal harms and consequences such as discrimination, the implications of confronting incidental findings, and concern about a lack of control without specific consent.

***Question 3b (If YES TO 3a): If sharing your genomic information in this situation was certain to result in benefits, which risks, if any, would you be willing to take?***

**Fig. 8:**
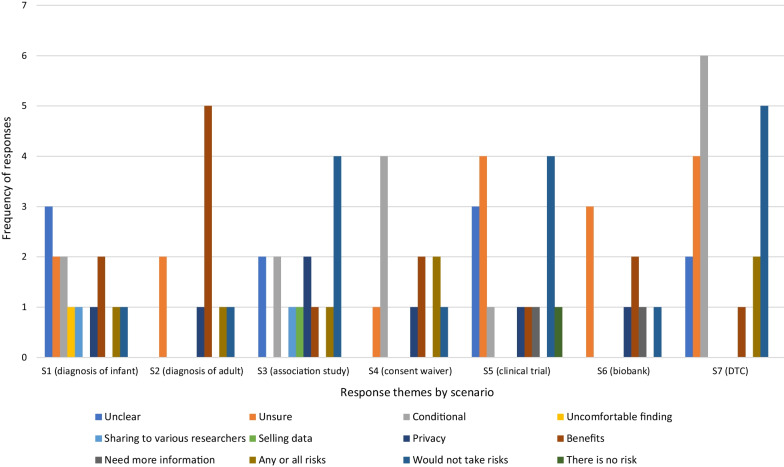
Distribution of themes for responses to *Q3. What risks would you be willing to take* by scenario

Themes indicating that respondents would not take risks, or would only take conditional risks appeared most frequently across the scenarios (Fig. 8). ‘Would not take risks’ was the strongest theme for S3 (association study) and S5 (private clinical trial), with respondents across several scenarios simply stating that “*I would never participate*” (Younger Man, S4), and that they would take “*no risk whatsoever*” (Younger Man, S6).

The acceptance of conditional risks, strongest in S4 (waiver of consent) and S7 (direct-to-consumer), was the most diverse of all the response categories in terms of its subthemes, which included conditions relating to consent “*I would generally agree to share it but would need a written contract setting out what is being shared for and how my identity would be protected*” (Older Man, S4), future use “*I would only risk sharing my genetic information if I was able to know where it was going and who would have access to it…*” (Younger Woman, S7), compensation “*I would need to be fairly compensated for me to give up this sort of information*” (Younger Man, S3), trust “*I will try to minimise the risk by carefully share[sic] the information only on trustworthy site*” (Younger Woman, S7), and regulation or protocols “*I would want to be across the terms and conditions*” (Younger Woman, S7).

An unusually high number of respondents indicated that they were unsure what risks they would accept; this theme was equal in total to those indicating acceptance of conditional risks. ‘Unsure’ was the most frequent response theme in both S6 (biobank) and S5 (private clinical trial) along with would not take risks in the latter. Responses for this theme were generally a straightforward ‘*I don’t know*” (Older Woman, S2), while others indicated a need for more contextual information “*not sure would have to know the risk first*” (Older Woman, S5).

***Question 4: Please describe the most important things that could be done in this situation to make you feel more comfortable about sharing if you were the patient/parent/research participant***

**Fig. 9:**
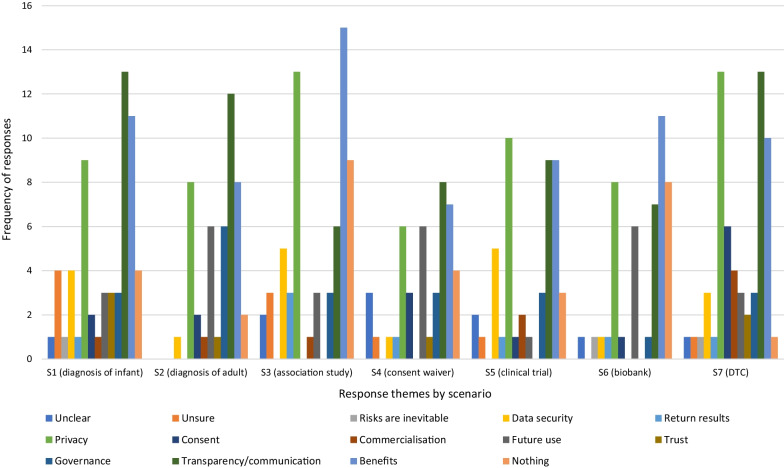
Distribution of themes for responses to *Q4. What measures would make you more comfortable sharing data* by scenario

The prospect of benefits was consistently strong across all scenarios (Fig. 9), and the most important measure to increase comfort in sharing for participants in S3 (association study) and S6 (biobank). Most comments emphasised the scientific and clinical benefits that may arise from sharing genomic information, for example, “*your DNA is being used for the good benefit of humanity. It will help improve everyone’s quality of life and may solve diseases that have lingered for years*.” (Younger Man, S7). Personal benefits were mentioned in the clinician-led scenarios (S1, S2, S3) and citizen-led sharing in the direct-to-consumer scenario (S7), for example that comfort in sharing would increase “*if there are any benefits for me in health*” (Younger Man, S3), however these were less frequent than benefits for all.

Transparency and communication was the most important factor to respondents in S1 (diagnosis of infant), S2 (diagnosis of adult), and S4 (waiver of consent), while in S7 (direct-to-consumer) this factor was equal to privacy as a measure to increase comfort. Respondents emphasised the need for “*transparency at all stages, including information on the risks (including likelihood and consequences) and benefits*” (Older Man, S1), with several respondents detailing the desire for clear communication around specific practices and protocols in data security, future use and the right to withdraw, among others.

Assurance of privacy was a strong precursor to feeling more comfortable sharing genomic information in all the scenarios, and was the most important factor for respondents in S5 (private clinical trial) and S7 (direct-to-consumer), equal with transparency/communication in the latter. Comments in this theme generally emphasised the importance of anonymity: *“…I do feel that if people could be reassured that their personal details would be secure that would be sufficient to ease any misgivings they may have*” (Older Woman, S4), and confidentiality measures providing “*reassurance that personally identifying information is not what the researchers are focussed on*” (Older Woman, S3).

Themes around governance, future use, and data security, appeared across all scenarios though with less frequency, as did the theme ‘nothing’, which was particularly strong in S3 (association study) and S6 (biobank). In this theme responses largely indicated that no further measures were needed as the respondents: “*feel comfortable enough about it already*” (Older Woman, S3), although for a small number the inverse was true in their scenario: “*I honestly don’t think there is anything that could make me feel comfortable*” (Younger Woman, S6).

## Discussion

In this study, the high rate of intention to share genomic data is striking in comparison to findings on intention to share in other recent investigations; for example, less than half of the Australian sample in the *Your DNA Your Say* study reported a willingness to share genomic data [25]. It is not clear at this stage how this difference can be accounted for; it is possible that here the use of prototypical scenarios provided a level of real-world grounding and detail that allowed respondents to contextualise their participation with greater confidence.

This research reinforces previous findings that members of the public tend to value broad social benefits more than specific personal benefits [26]. The importance of benefits to participants in genomic research has been reported in a number of empirical studies, particularly in the context of attitudes towards participation in biobanking, which involves storage and sharing of tissue, genomic information and other health information [26–28]. It has been noted, for example, that in this context people tend to be less concerned about privacy and confidentiality and more about who might benefit [27, 28].

Findings on intention to share also highlight the limitations of investigating public responses to genomic data sharing without accounting for the setting in which sharing occurs; while the overall mean intention to share is high in this study, it masks the dramatic variation between the lowest and highest rates of intention to share between the individual scenarios. The high degree of convergence both in the importance of benefits, and in the main types of benefits identified by respondents regardless of scenario suggests that the differentiation in intention to share between the scenarios may lie in the perceived risks, and the extent to which measures reported to increase participant comfort are addressed.

The diverse responses to risk in this study indicate that not only does intention to share vary across different scenarios, but that the factors that influence this intention vary in response to the context in which sharing takes place, highlighting the importance of further examination into how regulatory responses to genomic data sharing can account for public understandings and assessment of risk. Responding to generalized assumptions about risk and benefit without understanding the concerns specific to the sharing context may entrench the development or perpetuation of regulatory responses that are inappropriate or ineffective in fostering participant confidence, and thus successful data sharing practices. For example, in Australian research review procedures, risk assessment is almost wholly concentrated on personal risks to the individual participant; almost no account is taken of perceived societal harms (such as racial discrimination, commercial exploitation), and the impact this may have on participant consent in specific scenarios. This has the potential to be particularly problematic for the secondary use of data through waivers of consent, where research ethics bodies make decisions about the secondary use of genomic data on behalf of participants within this limited framework of assessment.

Similarly, in the two scenarios involving private industry (S5 and S7), which reported significantly lower intention to share and more frequent perceptions of risk compared to the other scenarios (see Table 3), some respondents indicated that it was not the act of sharing itself that troubled them necessarily, but a suspicion of the motivations of commercial actors and the future consequences, both broad and specific, that may eventuate from sharing their data. For these respondents, participation in scenarios involving private industry was perceived as a zero-sum game in which perceived commercial exploitation inherently undermined potential personal or public benefits. By contrast, fears of commercial exploitation and societal harms are less prevalent in Scenarios 3 and 6, which largely fit into more widely accepted conceptions of genomic research that are more likely to be perceived as altruistically motivated and serving the public good [17]. This finding is consistent with previous research around public perceptions of commercial involvement in genomic data sharing and biobanking, which have similarly identified lower intention to share with, and greater suspicion of, commercial stakeholders [16, 17, 25]. The persistence of this finding both locally and internationally suggests an intrinsic discomfort or “natural prejudice” [29] regarding private enterprise in genomic data sharing that presents ongoing challenges for a research environment increasingly intertwined with commercial interests and involvement [30, 31].

We are particularly interested in further investigating the consistent presence of future use in respondent risk perception. Though it appeared in differing levels across the scenarios, it was the most frequently reported risk overall (equal to privacy), indicating that concern about future use is common to members of the public regardless of the sharing context. In this study concerns about future use were not so related to malicious misuse—though this does appear—but the *unknown* quality of future uses and actors that, while possibly legitimate within the ethical and regulatory frameworks that govern GDS, may nonetheless conflict with the respondents’ values, priorities and thresholds for comfort. This presents a particularly complex regulatory challenge, given that it this same unknown quality of secondary use that informs current arguments towards open and broad consent practices in genomic data sharing [32]. It appears from this preliminary investigation that greater transparency around future use, along with clear communication of both benefits and regulatory safeguards are likely to contribute to ameliorating this concern; the character and relationship of these factors is likely to be a significant feature of subsequent investigation in this project.

### Limitations

As an exploratory investigation with a relatively small sample size the study has some inherent limitations. For example, the sampling characteristics were not exhaustive and do not represent every population sub-group that may have unique perspectives on GDS, nor does the analysis attempt to examine the intersection of both the participants' personal and social context and the context in which data sharing occurs. Further, despite the affordances of recruitment efficiency, the degree of opacity in participant selection through paid research panel operations can make it difficult to account for potential sampling biases. In this instance, a sample drawn from a paid research panel cohort may reasonably be expected to display a greater comfort in participating in other types of research; participants in this study did show a greater willingness to share genomic data compared to similar studies, though it is not clear if this finding can be explained by the recruitment method.

While the between-groups design of the survey facilitates a comparison of responses between the scenarios, it does potentially limit analysis compared to a within-groups design in which each participant would respond to all seven scenarios. However, given the technical complexity and length of the scenarios we determined that a between-groups design would minimise the likelihood of attrition and participant fatigue while still facilitating useful comparative data. That said, the depth of the written survey responses are likely to be limited in comparison to more robust qualitative methods such as semi-structured interviews. This is reflected in the brevity of some responses in the open-ended questions, which despite the maximal character allowance had a mean count of 19 words per response. However, as a preliminary study, the initial findings do provide insight and justification for further scenario-based investigations that are likely to generate a richer dataset for deeper analyses and recommendations.

## Conclusion

In this study we sought to initiate an exploratory qualitative investigation into how members of the Australian public respond to genomic data sharing in different sharing contexts, using validated prototypical scenarios. Our findings indicate observable patterns and diversity among the themes found within and between different sharing scenarios, as well as key themes that converge across the scenarios. These findings suggest that public responses to genomic data sharing are more complex than might be indicated by broad empirical research that does not take context of sharing into account; in particular, that the landscape of perceived risk is more diverse and contextual than might otherwise be understood without the comparative scenario-based analyses. These initial findings indicate the need for further investigation to explore public responses to and expectations of different GDS contexts in more depth, particularly if such analyses may contribute to the development of contextually appropriate recommendations for regulatory approaches.

## Supplementary Information

**Additional file 1.** Title: Full text of validated scenarios used in survey. Description: Full descriptive text of stakeholder validated prototypical surveys that were presented to survey respondents.

**Additional file 2.** Title: Full survey instrument. Description: Full text of the survey instrument presented to respondents.

**Additional file 3.** Title: Demographic distribution by scenario. Description: Presents demographic characteristics of the participant sample for each scenario.

**Additional file 4.** Title: Glossary of results – Themes and sub themes. Description: Glossary of themes and subthemes including example quotes.

**Additional file 5.** Title: Participant characteristics. Description: Full breakdown of participant characteristics as (n) and (%) within the whole sample.

## Data Availability

The datasets generated and analysed during the current study are available from the corresponding author on reasonable request.
