# Supplementary material for: Context matters in genomic data sharing: a qualitative investigation into responses from the Australian public
Source: BMC Med Genomics. 2023 Apr 1;15(Suppl 3):275. doi: 10.1186/s12920-023-01452-8 (PMC10068139; doi:10.1186/s12920-023-01452-8)
Supplement: Supplementary file 1 — Additional file 1. Title: Full text of validated scenarios used in survey. Description: Full descriptive text of stakeholder validated prototypical surveys that were presented to survey respondents. [file 12920_2023_1452_MOESM1_ESM.docx]

**Scenarios:**

1. A doctor seeks to diagnose and treat an infant patient with a suspected rare genetic condition. In order to do so, the doctor collects detailed medical information from the parent, and arranges an accredited laboratory for whole genome sequencing to be undertaken. The doctor has obtained written consent from the patient’s parent to obtain and analyse the medical information and whole genome sequence, and also to return relevant results to the family. The consent form includes a statement that “your information may be shared with others where necessary for diagnostic purposes”.

Further information/assistance is needed to confirm the diagnosis, so the doctor makes the decision to consult with other doctors who they already know and trust. In order to provide the best chance for a diagnosis, the information shared includes the patient’s medical records and relevant parts of the patient’s whole genome sequence. Personal identifiers like name, date of birth and address are removed from the medical files and files containing the genomic information. All information is stored on the doctor’s work computer network.

Although the information does not include identifiable information, with a lot of effort there is a very slight chance that it could be re-identified because the genomic information can only belong to one individual unless they are an identical twin. Sharing information about the patient is achieved face-to-face as well as through sharing files via the hospital’s computer network system, and sending emails. No formal arrangements or contracts are made between the doctors who share the information. The doctor returns the results to the patient’s parent.

1. A doctor seeks to diagnose and treat a patient with a suspected rare genetic condition. In order to do so, the doctor collects detailed medical information from the patient, and arranges an accredited laboratory for whole genome sequencing to be undertaken. The doctor has obtained written consent from the patient to obtain and analyse the medical information and whole genome sequence, and also to return relevant results to the patient. The consent form includes a statement that “your information may be shared with others where necessary for diagnostic purposes”.

   Further information is needed to confirm the diagnosis, so the doctor makes the decision to consult with other doctors who they already know and trust. In order to provide the best chance for a diagnosis, the information shared includes the patient’s medical records and relevant parts of the patient’s whole genome sequence. Personal identifiers like name, date of birth and address are removed from the medical files and files containing the genomic information. All information is stored on the doctor’s work computer network.

   Although the information does not include identifiable information, with a lot of effort there is a very slight chance that it could be re-identified because the genomic information can only belong to one individual unless they are an identical twin. Sharing information about the patient is achieved face-to-face as well as through sharing files via the hospital’s computer network system, and sending emails. No formal arrangements or contracts are made between the doctors who share the information. The doctor returns the results to the patient.

1. A hospital doctor also has a research position at the local university. They have a large collection of medical information and tissue samples from patients that are left over from medical care. The patients have agreed that these information and samples can be used for general research purposes. This means that the patients have agreed to allowing different researchers (including those in other countries) to use their information for a variety of future studies, and will not know which researchers will use their information and for what particular purpose. The doctor also has the same types of medical information and tissue samples from a group of healthy research participants, who have also agreed for these to be used for general research purposes. Personal identifiers like name, date of birth and address have been removed from all of the information files and tissue containers.

   The doctor has been using the information and samples for a research project, which includes obtaining genetic information from the samples through a technique called genotyping. Genotying involves testing whether small fragments of the whole genome that have been shown to be closely associated with particular diseases are present. To save money, the doctor engages an overseas laboratory to conduct this test.

   The doctor is a member of an international consortium that is coordinating the collection and analysis of genotyping and medical information for large studies that include enormous amounts of information from typically thousands of participants across multiple countries. The doctor wants to share some of their genotyping and medical information with the consortium for a collaborative research project. Although the information has been stripped of personal identifiers, with a lot of effort there is a very slight chance that it could be re-identified because the genomic information can only belong to one individual unless they are an identical twin. The genomic and medical information will be shared to other consortium members through a cloud-based platform. The members will only be able to view and analyse the data within this system, meaning they cannot copy the data to their own computers and use their own software to analyse it. The doctor has approval from their university ethics committee to participate in the research project. General findings of the research will be shared with participants through regular newsletters, but there is no provision for sharing of individual results.
2. A cancer researcher has a tissue bank of pancreatic cancer tumours and surrounding normal tissue which were collected from Indigenous Australians. Whole genome sequencing was performed on both the tumour and normal tissue in 2013. The researcher now wants to publish the results of the research in a prestigious scientific journal, but the journal requires that the whole genome sequence data is deposited in a public international data repository The repository allows only approved researchers to access and analyse the information. A data access committee imposes restrictions on who can access the deposited information and carefully assesses and then approves or denies access to the data. The information will be shared to approved researchers through a cloud-based platform Although the information has been stripped of personal identifiers, with a lot of effort there is a very slight chance that it could be re-identified because the genomic information can only belong to one individual unless they are an identical twin.

The original participants who donated their tissue agreed to allow the original researcher to keep it for use in further research on pancreatic cancer. The consent did not say anything about sharing their data for other purposes. It also did not mention whether any individual results of any future research would be returned to participants. The researcher has decided that it will be really difficult and impractical to recontact the participants to obtain their consent to share their information, especially since the researcher estimates that half of the participants are likely to have already passed away. The researcher therefore gets approval from their university ethics committee to waive the requirement for consent to share the anonymous genomic data. This means the ethics committee would make the decision as to whether the researcher can share the genomic data without the approval of the original research participants. A US-based research team gets approval from the repository’s data access committee to use the data for research and identifies a small number of participants who possess a particular gene that may increase their risk of a serious disease that could be preventable. That is, if the individual knew they were at risk, they could alter their lifestyle or take medications known to decrease the likelihood that the disease will occur. The US research team contacts the original researcher to let them know.

1. A researcher recruits participants with a known condition/diagnosis for a clinical trial of a new and original treatment/medication. The trial is funded by a private for-profit company. As a part of the trial, participants will need to provide blood samples for genotyping. This involves obtaining genomic information from the blood by testing whether small fragments of the whole genome are present that have been shown to be closely associated with particular diseases.

   The blood samples are stored in a centralised biobank operated by the same company funding the clinical trial. The biobank maintains a database of genomic information that is linked to the individual donors’ medical information. Personal identifiers like name, date of birth and address have been removed from all of the information files and blood sample containers. Although the information has been stripped of personal identifiers, with a lot of effort there is a very slight chance that it could be re-identified because the genomic information can only belong to one individual unless they are an identical twin.

   The biobank will be maintained by the company with unclear future storage and sharing practices. Participants may withdraw from the clinical trial at any time but the company states that this does not include the ability to withdraw from the future use of their samples since they will already have been added to the biobank in a de-identified form.
2. A team of Australian researchers have obtained whole genome sequence information from four large biobanks in the U.S., Europe and Australia for a research project. The researchers have been able to get access to the data by applying to each biobank’s data access committee. The Data Access Committee will only release data where it assesses the sharing as being within the scope of the original participant consent. For example, if the original donor consented to allowing their genomic information being used for cancer research and the researchers wanting access are conducting research into something else (e.g. diabetes) the data access committee will not allow them access.

   Before the data is released, the approved researchers must enter into a data transfer agreement with each of the biobanks. Although the Data Transfer Agreements are not identical, each includes conditions on use and subsequent sharing, including an obligation not to re-identify individual donors. Personal identifiers like name, date of birth and address are removed from the genomic results. Although the information does not include identifiable information, with a lot of effort there is a very slight chance that it could be re-identified because the genomic information can only belong to one individual unless they are an identical twin. Some of the source biobanks have technical solutions to avoid re-identification. For example, they may have computer programs to mix up the data so that they are only interpretable by those who have access to the program.

   Donors have given broad consent for the use of their genomic sequence information. Therefore the donors have agreed to allow their genomic information to be used for general research purposes. This means that the donors have agreed to allow different researchers to use their information for a variety of future studies, and will not know which researchers will use their information and for what specific purpose. Thus, they will not know that the data has been provided to this specific research team. The research team has no way of getting in touch with the original donors.
3. A woman has been diagnosed with a genetic condition. She is keen to find out more about her condition, and has sent a saliva sample to a direct-to-consumer genetic testing company, whose reports include information relating to health risks. She uploaded her genetic report and her (self reported) associated clinical symptoms to an online “Match My Genome” site. In doing so, her aim was to discover more about her diagnosis and find a community of people with related conditions. Through the site, people get real-time information about scientific advances and talk with others to share experiences. Scientific advances generally include the results of new studies that discover the genetic cause of disease, mental health conditions or other attributes (e.g. sporting ability). The site uses false names meaning contributors are not identifiable, but the site provides a “private message” function. This means that people using the site can contact each other privately.

   The use of data after it has been uploaded is governed by the site’s terms and conditions, which users may or may not read or understand. Some of the companies allow other research organisations to access this data and use it for research purposes, both with and without the consent of the original customers. The site has no restrictions on the development of future intellectual property from uploaded data. This means someone can use the uploaded genomic information to invent a new drug, for example, without acknowledging whose genomic information they used.
