# Supplementary material for: Context matters in genomic data sharing: a qualitative investigation into responses from the Australian public
Source: BMC Med Genomics. 2023 Apr 1;15(Suppl 3):275. doi: 10.1186/s12920-023-01452-8 (PMC10068139; doi:10.1186/s12920-023-01452-8)
Supplement: Supplementary file 2 — Additional file 2. Title: Full survey instrument. Description: Full text of the survey instrument presented to respondents. [file 12920_2023_1452_MOESM2_ESM.docx]

# Australian attitudes towards sharing DNA data

**The purpose of the survey?**

This survey aims to assess Australians' views about how health data is shared. We are interested in what people think about the sharing of genetic and genomic data between different professionals and organisations for different purposes. Sharing this data is a way to make the most of this important information and potentially speed up the discovery process. It is also a way of improving the ability of doctors to diagnose and treat patients.

At the moment how and why scientists and doctors are sharing genetic data differs. There are also varying policies and procedures that need to be followed depending on the type of organisation wanting to share or access data, the type of health professional, and the country (or state) in which they live.

The aim of this specific survey is to find out what members of the general public think about this situation so that we can incorporate your views into recommendations and reforms designed to protect donors and also to help scientists and doctors.

**What does the study involve?**

Participation involves watching a brief video about genetic information, reading a scenario describing a typical data sharing situation, and answering open ended questions about the scenario. You will also be asked about your demographic background.

**What is the time commitment?**

The survey should take approximately 20 minutes to complete.

**Will all data provided be confidential?**

Your participation in this study is completely voluntary and you are free to exit the survey at any time. All responses will remain confidential. If requested by peer reviewed journals or external researchers, completely de-identified summaries of the data may be shared. This data will in no way be able to identify you and will be used for research purposes only.

**Should participation in this study cause distress:**

It is not anticipated that this survey will raise any distressing issues. If any problems arise, you can call Lifeline Australia on 13 1114 (24 hours) or access support online.

# Who is conducting the study?

The University of Tasmania: Professor Diane Nicol and Dr Rebekah McWhirter

Swinburne University: Professor Christine Critchley and Mr Jarrod Walshe.

# Questions about the study:

If you have any questions regarding the survey, please contact:

Prof Christine Critchley

Swinburne University
(03) 9214 5480
ccritchley@swin.edu.au

# Research ethics:

This project has been approved by or on behalf of Swinburne and the University of Tasmania's Research Ethics Committees in line with the National Statement on Ethical Conduct in Human Research.

If you have any concerns or complaints about the conduct of this project, you can contact:

Research Ethics Officer
Swinburne University of Technology
PO Box 218 Hawthorn VIC 3122

(03) 9214 3845
[resethics@swin.edu.au](mailto:resethics@swin.edu.au)

Please click here to download a more detailed version of the Participant Information Statement for your records.

Do you consent to participate in the Australian attitudes towards sharing DNA data survey?

– No, I do not wish to participate

– Yes, I consent to participate

We care about the quality of our survey data. This is an important project that may inform Australian public policy regarding the sharing of DNA data.

Please note that during the survey you will be asked to watch a two-minute video and to carefully read a detailed scenario involving the sharing of DNA data. **If you do not take the time to watch the video or read the scenario you will not be eligible to complete the survey.**

Before you begin will you give your commitment to give the survey your full attention and to thoughtfully provide your best answers to the questions?

– I will give my full attention and provide my best answers

– I will not give my full attention or provide my best answers

**Demographics**

We would like to ask some questions about you to help us ensure we receive the views of different groups of people.

**1.** What is your gender?

– Male

– Female

– Prefer to self-describe as: ______________________

**2.** What is your age group?

– Under 50 years of age

– 50 years of age or over

**3.** What is the **highest** level of education you have completed?

– Below year 12, including Certificates I & II

– Year 12 or equivalent

– Certificate III, IV, or Diploma

– Some University

– University undergraduate degree

– University postgraduate degree

**4.** Have you ever been diagnosed with a serious health condition? By serious we mean a health condition where there is no known cure, it is ongoing, and decreases the quality of your life.

– Yes

– No

– I don't know

**5.** Have any of your immediate family members been diagnosed with a serious health condition? By serious we mean a health condition where there is no known cure, it is ongoing, and decreases the quality of your life.

– Yes

– No

– I don't know

**6.** Do you identify as Aboriginal and/or Torres Strait Islander?

– Yes

– No

**7.** Which ethnic or cultural background do you most identify with?

– Australian

– Another background

**8.** Were you born in Australia?

– Yes

– No

**9.** What is your postcode

__ __ __ __

**10.** Would you describe where you live as urban or rural/remote?

– Urban

– Rural (or remote)

**11.** Do you have children?

– No

– Yes

**Video**

Before we start the survey we want to make sure you are familiar with some important concepts such as genomes, genes and sequences. The following 2-minute video explains these concepts. Please watch the video and then we will ask you some brief questions to make sure you are aware.

Embedded YouTube video: <https://www.youtube.com/watch?v=ictAm2wSwtY>

To make sure that you are familiar with the key aspects we will cover in this survey please answer the following statements. For each statement please indicate whether you believe each statement is true or false.

| A gene is a small section of the genetic sequence that relates to a particular characteristics of the person | TRUE | FALSE |
| --- | --- | --- |
|  |  |  |
| The order of a genetic sequence can determine how our bodies look and work | TRUE | FALSE |
|  |  |  |
| A person's genome contains the entire sequence of DNA letters from all 46 chromosomes | TRUE | FALSE |
|  |  |  |
| Sequencing or testing the whole genome provides scientists or doctors with a person's entire genetic code or information | TRUE | FALSE |
|  |  |  |
| A specific genetic test or sequence provides scientists or doctors with the information relating to only an individual's specific characteristic (e.g. risk of disease, hair colour). | TRUE | FALSE |
|  |  |  |
| A genetic sequence is the order in which the DNA letters (or chemicals) occur across the strands of our DNA | TRUE | FALSE |

# Scenario Presentation

It is important that you read the scenario on the next page very carefully as all of the following questions will be based on it. You will be able to view the scenario at any time while answering the questions. The scenario may contain unfamiliar terms or concepts. These are highlighted in blue and contain further information. If you are unfamiliar with them just click on the text and you will be provided with an explanation. To remove the pop-up text, click anywhere again.

| SCENARIO |
| --- |

We are now interested in your thoughts about this data sharing scenario. Please answer the following questions, imagining that it is your genetic information that is being shared.

At any time you can press the 'View Scenario' button and the scenario will appear again for you to read. To hide the scenario, click the button again.

**S1.** If you were the patient in this scenario, would you be happy for the doctor to share your results?

**–** Yes

**–** No

**–** Depends

(If yes S1) Please describe why you would be happy for the doctor to share the results of your whole genome.

|  |
| --- |

(If no S1) Please describe why you would not be happy for the doctor to share the results of your whole genome.

|  |
| --- |

(If depends S1) You have indicated that your decision on whether or not you would be happy for the doctor to share the results of your whole genome depends. Could you please describe why?

|  |
| --- |

**S2.** If you did to decide to share your genomic information in this scenario, do you think there would be any benefits or positive consequences?

**–** Yes

**–** No

(If yes S2) Please describe what you think those benefits or positive consequences could be.

|  |
| --- |

(If no S2) Please describe why you think there would be no benefits or positive consequences.

|  |
| --- |

**S3.** If you did to decide to share your genomic information in this scenario, do you think that there would be any risks or negative consequences?

– Yes

– No

(If yes S3) Please describe what you think those risks or negative consequences could be.

|  |
| --- |

(If yes S3) If sharing your genomic information in this situation was certain to result in benefits, which risks, if any, would you be willing to take?

|  |
| --- |

(If no S3) Please describe why you think there would be no risks or negative consequences.

|  |
| --- |

Finally, we are interested in what you think is needed to make people feel more comfortable about any potential risks associated with sharing genomic information. Please describe the most important things that could be done in this situation to make you feel more comfortable about sharing if you were the patient.

|  |
| --- |
