# Supplementary figures and images for: Context matters in genomic data sharing: a qualitative investigation into responses from the Australian public
Source: BMC Med Genomics. 2023 Apr 1;15(Suppl 3):275. doi: 10.1186/s12920-023-01452-8 (PMC10068139; doi:10.1186/s12920-023-01452-8)

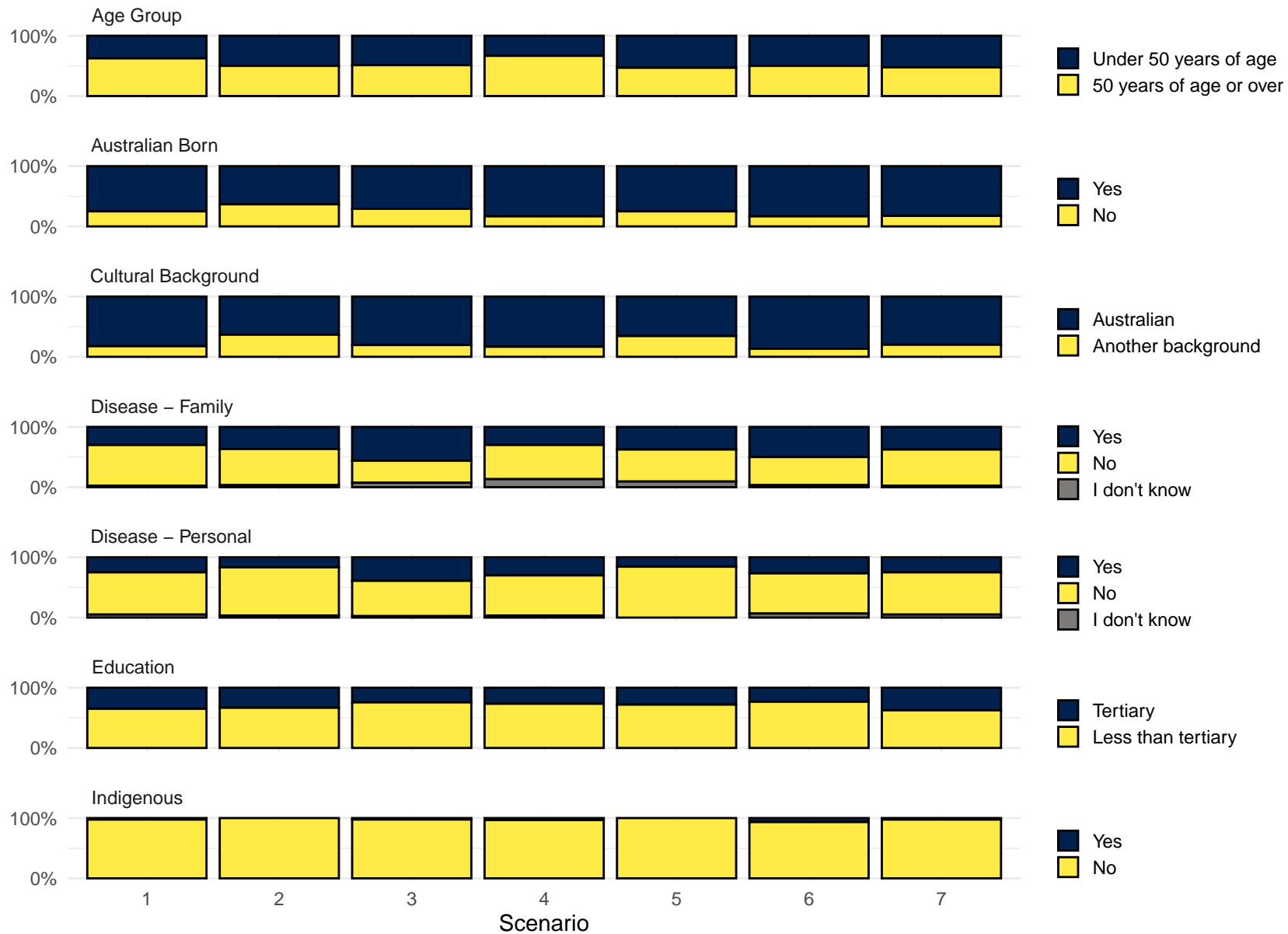

Supplement: Supplementary file 3 — Additional file 3. Title: Demographic distribution by scenario. Description: Presents demographic characteristics of the participant sample for each scenario. [file 12920_2023_1452_MOESM3_ESM.pdf]
