# Supplementary material for: Context matters in genomic data sharing: a qualitative investigation into responses from the Australian public
Source: BMC Med Genomics. 2023 Apr 1;15(Suppl 3):275. doi: 10.1186/s12920-023-01452-8 (PMC10068139; doi:10.1186/s12920-023-01452-8)
Supplement: Supplementary file 4 — Additional file 4. Title: Glossary of results – Themes and sub themes. Description: Glossary of themes and subthemes including example quotes. [file 12920_2023_1452_MOESM4_ESM.docx]

**Summary of results – Themes and sub themes**

**Part one – Definitions and example quote(s) for all themes**

**Question 1: If you were the (Patient/Parent/Research participant), would you be happy for the (doctor/researcher) to share the results?**

**Codes for YES -** Please describe why you would be happy for the (target) to share the results of your (your child’s) whole genome?

| **Theme** | **Definition** | **Subtheme** | **Example quote(s)** |
| --- | --- | --- | --- |
| **No Concerns** | Participation is based upon the general idea that there are no concerns, worry or harm involved with sharing one's data |  | I don’t see any harm in it. |
| **Benefits** | A belief that sharing will benefit the wider community by increase the likelihood that science will advance in terms of discovery and the ability to predict and data prevent disease, accuracy in diagnosis will increase and new or improved treatments and cures will be developed. General and specific benefits to particular groups (children or those with rare disease) were mentioned. | General benefits | If I can help with this in any way - if anyone else can be helped, then I'm all for it. |
|  |  | Scientific discovery/research | if it can help the scientific community in accumulating more knowledge in regard to genetics and how they can contribute to diseases and then perhaps in the future find cure or prevent illnesses (person 1). I believe sharing these results may help doctors identify the likelihood of disease and could result in preventative action being taken (person 2) |
|  |  | Diagnosis | It would help in the treatment and diagnosis of medical problems now and in the future. |
|  |  | Cures and treatment | Sharing this type of information around the world will hopefully lead to a quicker resolution of finding causes and cures for diseases. |
|  |  | Children | any effort to help a child is welcome |
|  |  | Rare diseases | it is due to trying to find an appropriate treatment or way forward for a rare medical condition |
| **Personal benefit** | Participation is based on a desire for benefits to the self or one’s children, including treatments or better diagnosis/awareness both now and in the future. Comments also reflected individual value in obtaining information for personal use including convenience, ownership of data, curiosity and ability to communicate knowledge of one's genetic information to others. | General/Health benefits | It would give me the best chance for a cure |
|  |  | Information/prevention | I would be pleased to learn about anything that would improve and extend my journey through my senior years. |
|  |  | Ownership | I don't see a piece of skin flake or a discarded toenail as "myself", sure It came from me, and the DNA was mine, but I don't care enough to take ownership over it. - small things like this shouldn't matter. |
|  |  | Convenience | no inconvenience to me if they already have the DNA |
|  |  | Communication | because I can tell my story |
|  |  | Curiosity | it would be interesting to see the result they produce from my genes |
| **Collaboration** | A specific belief that collaboration will improve outcomes and efficiencies resulting in increased knowledge, discovery, diagnosis and treatment. This includes the idea that sharing different types of information would be beneficial and not reusing data is inefficient |  | It is only by providing and sharing information that medical advancements and cures can progress (person 1). If I was contributing to science, I would expect the data to reusable (person 2). |
| **Privacy** | The perception that there would be limited privacy risks, or that the benefits of genomic research outweigh privacy risks. Also, some, views represented the idea that participation would depend upon privacy protection. | No or low risk | All personal identifiers are removed so I don't feel there is any invasion of privacy. |
|  |  | Benefits out weigh risk | I have nothing to hide and am beyond worrying about such things being intrusive. The betterment of science is more important than my stance on privacy. |
|  |  | Conditional on privacy protection | I wouldn't mind if it helped science, however, I would need to be assured that I remained anonymous to the general public |
| **Trust** | The perception or belief that the doctor/researcher, data protection process or whom the information is shared with can be trusted. | Researcher/doctor | I would like to think that a doctor always has my (or others) best interests at heart |
|  |  | Security of data | I trust the security of the information as it would not feasibly be possible to identify my child |
| **Consent** | A belief that sharing is acceptable as long as consent has been obtained. |  | I wouldn't have agreed to the study in the first place if I were not happy with the terms. |

**Question 1: If you were the (Patient/Parent/Research participant), would you be happy for the (doctor/researcher) to share the results?**

**Codes for No** - Please describe why you would not be happy for the (target) to share the results of your (your child’s) whole genome?

| **Theme** | **Definition** | **Subtheme** | **Example quote(s)** |
| --- | --- | --- | --- |
| **Future Use** | Concerns about applications that may be outside of the original purpose and a lack of awareness around who may access the data and/or for what purpose. | Outside of original purpose | unsure what the researchers may do with my genome once shared other than the clinical trial, it's quite discomforting not knowing where it may go and for what other purpose (person 1). What are they going to do with my DNA? Build clones? (Person 2) |
|  |  | Awareness | Because there is no information about what the future use of this is (Person 1). Without further clarification on how the information will be shared into the future, I would rather err on the side of caution (Person 2). I would want to have clear and written explanation of who, what, where and why my genetic information would be used (Person 3) |
| **Privacy** | Concerns that data could be identified and that mechanisms designed to protect privacy such as re-identification procedures are either not present or inadequate. These comments also include general statements indicating the importance of privacy. |  | My privacy and that of my family come first (Person 1). I don't want anything shared that could identify me (Person 2). I do not believe that technology is safe enough to keep personal information separate and non identifying (Person 3) |
| **Consent** | A desire to be asked permission before information is shared in general or a desire to be asked to consent to specific uses of information. | General | I would want to have clear and written explanation of who, what, where and why my genetic information would be used. I would not want my personal, genetic information shared with outside sources without my permission and consent. |
|  |  | Specific consent | Because I would have made an agreement (effectively a contract) that my data is used only for a specific purpose. I should be recontacted if this needs to change. |
| **Commercialisation** | Concern associated with the commercial use of information in general, the idea that genetic information is owned by the self and should not be sold, and that commercialisation may inhibit access to those in need. | Commercial use | I don't believe that my DNA should be used for commercial purposes that I am unaware of. |
|  |  | Access | While it may assist the development of ground-breaking drugs, these could be priced too high by big pharma to be of any use to those who most need the drug and whose info may have contributed to the discovery in the first place. |
|  |  | Ownership | its my information. what is stopping the company form selling that information |
| **General concern/fear** | This represented one comment that expressed fear |  | I'm scared |

**Question 1: If you were the (Patient/Parent/Research participant), would you be happy for the (doctor/researcher) to share the results?**

**Codes for Depends -** You have indicated that your decision on whether or not you would be happy for the (target) to share the results of your (your child’s) whole genome depends. Could you please describe why?

| **Theme** | **Definition** | **Subtheme** | **Example quote(s)** | | |
| --- | --- | --- | --- | --- | --- |
| **Consent** | Sharing depends on consent being obtained |  | If the people didn't consent, they didn't consent. It's as simple as that. If it were me, I would at least like to be asked first. | | |
| **Commercialisation** | The desire to share depends upon the information not being used for commercial interest in general and specifically for insurance or employment purposes. Also a view that the individual owns their genetic material, it should not be patented and if profits are made they should be returned to the owner. | General | If they use it for develop medicine to help other people who has similar situation with me I'm more than happy to share it but if they use it for business I'm not happy | | |
|  |  | Ownership | Some companies, especially in the US, have patented genetic sequences. How this is at all legal is beyond me. | | |
| **Benefits** | Sharing depends on whether or not it will be beneficial and the urgency of the need for these benefits | General | for me it depends on the situation if it would help yes if it's not necessary no | | |
|  |  | Self | If there was a chance that a drug could be invented to help with my disorder I guess my DNA could be used. | | |
|  |  | Others | If the drug was to help create a good drug for the benefit of humanity etc coronavirus, it would be ok. | | |
|  |  | Urgency | It depends how serious things are | | |
| **Future use** | Sharing depends on who the information is shared with and/or for what purpose | Who it is shared with | because it depends who is he sharing the information with | | |
|  |  | Purpose | If I originally said OK, then that is OK but if I did not and only wanted research say for cancer then it should not be shared in other fields. | | |
| **Awareness/knowledge** | Sharing depends on the provision of more information or greater awareness about the specific situation or context or reasons for why sharing is needed |  | I would need to be given the reasons as to why it is important for the results to be shared before agreeing. | |  |
| **Type of information/results to be shared** | Allowing access depends on the type of information or results to be shared. This includes what the test results indicate, the integrity of the results and whether the results have been manipulated |  | it depends what the test reveal | | |
| **Governance** | Sharing depends on the presence of general contracts, agreements or restrictions and/or specific regulations associated with security |  | maybe be okay if a contract a or guidelines created between doctor in sharing of information | |  |
| **Privacy** | Concern about privacy protection |  | because this data could be potentially linked to me |  | |
| **Security of data** | Sharing would depend on the knowledge that the data is protected and the possibility it can be shared even more widely |  | It would need to be confirmed that the information is protected (person 1). How far does this information go? Or is it possible that company may share it further? (person 2) | |  |
| **Harm** | Sharing depends on whether harmful or unethical consequences (in general or in relation to specific harms) will occur and possible negative implications for family members as well as the self. Specific consequences include fear of future discrimination by employers or insurance companies. |  | what the consequences are (Person 1). would it affect my descendants/siblings opportunity to obtain reasonable cost life/health insurance, Or even their opportunity to obtain work in particular industries or areas of professionalism? might it cause isolation inability to gain a life partner? (Person 2) | | |
| **Trust** | Sharing depends on trust in those asking for use of the data and in those accessing the data |  | if the people handling the information could be trusted | |  |

**Question 2: If your (your child’s) genomic information was shared in this situation, do you think there would be any benefits or positive consequences?**

**Codes for Yes** - Please describe what you think those benefits or positive consequences could be.

| **Theme** | **Definition** | **Subtheme** | **Example quote(s)** |
| --- | --- | --- | --- |
| **Cures or treatments** | The scenario is likely to increase cures and/or treatments including new drugs for disease. Different applications are for the self and family members, or for all in general. | Self & Family | The doctor may find a cure to my disease (Person 1). it can benefit my future children or grandchildren or future generation of my immediate family (Person 2) |
|  |  | For all or in general | There may be no benefits to me, but perhaps to the greater community (Person 1). finding new treatments for potentially incurable diseases or conditions (Person 2) |
| **Diagnosis** | The scenario is likely to increase or improve diagnosis of disease. Comments reflected improved diagnosis in general (general statement that improved diagnosis will result) or specifically referred to the application to the self. | Self | a chance to find any faults that I carry |
|  |  | In general | because it would be easier to find out the origin of any abnormalities |
| **Awareness/knowledge** | The scenario will lead to an increase in awareness or knowledge beneficial to the self, families, the scientific and medical research community or not stated (general statement that knowledge or awareness will increase) | Self & Family | I may discover some important information about my body/ancestry (Person 1). Medical information for future generations of my family (Person 2) |
|  |  | Research | For scientists to fully understand diseases being transmitted thru' genes. |
|  |  | In general | I expect they would be able to learn new information or confirm previously gathered information by doing so. |
| **Prediction/Prevention** | The scenario is likely to lead to information from which future health (or other outcomes) can be made. This information may also be used for preventative purposes. | Self & Family | I would have information to help me make life decisions (Person 1). The positive benefits may be that the person in question's relatives may be alerted to the possibility of illnesses and therefore able to modify behaviour to prevent these diseases occurring (Person 2) |
|  |  | In general | Discovering genotypes for certain diseases, and other environmental factors that play into the development of these diseases, so they can be prevented in the future. |

| **Scientific method/Medical procedures** | The scenario will lead to improvements in the scientific method by increasing efficiency (saving time and resources) or efficacy (become more accurate and/or clinically valid) due to increased collaboration. Comments reflect simply collaboration as a benefit or relate it to increased expertise or resources (data sets). | Collaboration | It will help construct a database for identifying illnesses etc and possibly finding cures for it. |
| --- | --- | --- | --- |
|  |  | Efficiency | better use of time and resources to target the condition |
|  |  | Efficacy/Clinical utility | This is not seen as a case of "too many cooks....", but consultation with a number of experts in their field must surely return better results, and those results could not be obtained without the experts having all the information available to them |
| **Communication** | The scenario will lead to a greater ability to be able to communicate and share aspects of the self with others |  | it would allow others to identify with my situation (Person 1) because I can tell my story (Person 2) |

**Question 2: If your (your child’s) genomic information was shared in this situation, do you think there would be any benefits or positive consequences?**

**Codes for No** - Please describe why you think there would be no benefits or positive consequences

| **Theme** | **Definition** | **Subtheme** | **Example quote(s)** |
| --- | --- | --- | --- |
| **No personal benefit, but others will benefit** | Although respondents stated that they would not personally benefit, there was an acknowledgement that scientific research, those able to make a profit or other people may benefit. |  | I meant for me personally there may be some help for scientific facilities (Person 1) I wouldn't be getting anything out of it personally. The only positive consequence would be the value of the data for scientific research (Person 2). While there would be no benefits or positive consequences to me it might help someone else (Person 3) |
| **Commercialisation** | An acknowledgement that others would make money from selling or receiving information |  | not for me there wouldnt be but the person who is selling it is who would benefit by making money (person 1). It might not be beneficial to me as I might be charged for higher private healthcare in the future (person 2) |
| **No benefit** | No benefits were reported for the self, other people or in general | General | What is the point? Why are they studying random DNA? (Person 1). Genome sequence can't be changed so makes do difference in sharing it, and everyone's is different so wouldn't help others (Person 2) |
|  |  | Personal | It doesn't benefit me personally |
| **Illegal** | One respondent simply stated that the activity in the scenario was illegal so benefits did not matter. |  | It doesn't matter. It would be illegal. |
| **Lack of transparency** | A belief that purpose of use and therefore benefits are not transparent |  | I just think if someone can't disclose what this would be used for then there must be something not too good behind it, maybe not but this info is pretty important |
| **Accuracy/clinical utility** | Quality or usefulness of the data or the expertise involved in the scenario is questioned. |  | I would rather have the same service done through a medical institution |
| **Potential for misuse** | The belief that potential misuse of data could occur |  | Information derived from such studies could be skewed by governments or other powerful organizations to alter the basic human reproductive cycle we have known. |
| **Privacy** | Concerns about the possibility of privacy breaches due to vulnerability of technology to protect data or the sensitive nature of the data. |  | I don't want anything shared that could identify me. I do not believe that technology is safe enough to keep personal information separate and non identifying (Person 1). It could contain some serious information that should not be shared to anyone else (inheritance of disease, the factor that could emit genetic diseases) (Person 3) |

**Question 3a: If your genomic information was shared in this situation, do you think that there would be any risks or negative consequences?**

**Codes for Yes -** Please describe what you think those risks or negative consequences could be

| **Theme** | **Definition** | **Subtheme** | **Example quote(s)** |
| --- | --- | --- | --- |
| **Commercialisation** | Risks involve industry or business being involved to benefit financially from the information, selling information and IP rights | General | Business purpose, some people will take advantage to gain benefit (Person 1). The data could be used by medical insurers and companies in a general sense to identify risk factors and increase costs for those with specific genetic risks (Person 2) |
|  |  | Selling information | the potential for on selling data |
|  |  | IP | Grey area as to who has a share to the IP over any discoveries enabled by the information |
| **Future use** | Risks associated with future use included sharing with unknown others and for reasons outside of the original purpose, which can lead to the possibility that the information can be used misused or abused. |  | Unscrupulous use of the DNA (Person 1). Anything they decide to use it for that is against what I would agree with (Person 2). but you never know who’s hands it could end up in (Person 3) |
| **Privacy** | Risks that individuals or the self can be identified in general or that their sensitive and personal information will be known to others |  | I don't want anything shared that could identify me. |
| **Consent** | The possibility that the information could be used without consent |  | There is a possibility that personal, sensitive genetic information could be shared with outside sources without users' consent. |
| **Unauthorised access** | The possibility that the data may end up in the hands of unauthorised, unscrupulous or inappropriate people. This was also associated with the possibility of hacking or data theft and the chance that technology is not robust enough to mitigate these risks. | General | If information fell into the hands of inappropriate people |
|  |  | Data security | Online databases can be reasonably easily hacked (Person 1). I do not believe that technology is safe enough to keep personal information separate and non-identifying (Person 2) |
| **Harm** | Outside of commercialisation and unauthorised access other harmful consequences were mentioned. These include general harms such as embarrassment, errors, discrimination and specific consequences such as crime detection and bioweapons. |  | only if they tried to use my results a harmful way (Person 1). they can be used instead to develop bioweapons that would only target a certain race (Person 2). I suppose these is always the risk that it gets mixed up with someone else’s DNA or it gets into the wrong the hands (Person 3). Maybe if I become a serial killer sharing my genome now will get me caught in 30 years (Person 4) |
|  |  | Specific |  |
| **Incidental findings** | The risk that information about a potential health condition or parentage would be revealed to the self that may be unwanted or uncomfortable. | General | something I might know about myself which I don’t want to know |
|  |  | Health | possibly finding out about a condition that you or your family were not aware of, possibly a genetic condition |
|  |  | Paternity | My image as a father would be broken by everyone who knows the genetic information of my child. |
| **No or minimal risk** | Risks overall are viewed as minimal or do not exits. |  | I don't foresee any. I'm not worried about medical professionals discussing my information (Person 1). there could be 'risks', but I think they are VERY small and quite warranted when all weighed up (Person 2) |
| **Risks are not known** | The idea that there are unknown risks |  | well you never know |

**Question 3b: (if YES to 3a) If sharing your (your child’s) genomic information in this situation was certain to result in benefits, which risks, if any, would you be willing to take?**

| **Theme** | **Definition** | **Subtheme** | **Example quote(s)** |
| --- | --- | --- | --- |
| **There is no risk** | Respondents indicated a perception that there were no risks |  | I can see no foreseeable risks |
| **Would not take risks** | Limited or no risks would not be taken in general or if one's safety was compromised | General | none I wouldn’t give anyone or allow to take my DNA |
|  |  | Specific | Nothing that compromised my safety (Person 1) |
| **Any or all risks** | Statements reflect a willingness to accept all or most risks |  | I would accept the possibility of risk s of any kind |
| **Need more information** | The need for extra information before knowing what risk one is willing to take |  | I don't know what risks there would be as this is really sci fi type stuff to me. I would have to discuss it with my doctors and do some research to help me decide if I would release it or not |
| **Benefits** | Most or unspecified risks would be sacrificed if the probability of general health benefits or specific benefits to the self or children was higher than any risk | Relative to risk | As long as the probability of benefits occurring outweighs the probability risks happening, no matter the type of risk, I would be willing to share my genomic information |
|  |  | Personal/child | I would weight up the risks at the time and decide then, only if it were a certain benefit that would help me would I be happy to share (Person 1). Yes if it meant for the best results for the child (Person 2). |
| **Privacy** | Compromised or risks to privacy or confidentiality would be acceptable |  | If guaranteed to benefit someone, I would risk my privacy to a certain degree |
| **Selling data** | Allowing data to be sold if it were anonymous |  | The risk of my anonymously provided data to be on-sold or compromised by 3rd parties. |
| **Sharing to various researchers** | The risks associated with allowing unknown actors to access the data |  | I thought the only risk noted was in the possible sharing of information to other experts in the field. Thus the risk of it maybe getting seen by the wrong person. Most people likely would have no use for it. Anyway, that’s the risk as I understand it, so that sort of self-answers what risk I am willing to take. |
| **Uncomfortable finding** | The chance that a certain result would be challenging |  | Knowing the information that I could inherit diseases to my child. |
| **Conditional** | Willingness to take risks in general as long as certain conditions were in place such as consent, future use of data, compensation, trust and regulation or protocols | Consent | None, without my prior consent. |
|  |  | Future use | I would only risk sharing my genetic information if I was able to know where it was going and who would have access to it. |
|  |  | Privacy | If the information is kept strictly confidential and data destroyed after I would be more willing |
|  |  | Compensation | I would need to be fairly compensated for me to give up this sort of information |
|  |  | Trust | With my situation I can help others, but it will attract businessman to take advantage however I will try to minimise the risk by carefully share the information only on trustworthy site |
|  |  | Regulation/protocols | I would generally agree to share it but would need a written contract setting out what is being shared for and how my identity would be protected |

**Question 3a: If your genomic information was shared in this situation, do you think that there would be any risks or negative consequences?**

**Codes for No -** Please describe why you think there would be no risks or negative consequences.

| **Theme** | **Definition** | **Subtheme** | **Example quote(s)** |
| --- | --- | --- | --- |
| **No or negligible risk** | Respondents cannot see any harm or risks associated with the scenario in general. Some mentioned that the nature or value of genomic data itself prevents risks of identification or misuse. That is, the data is of no value to others and it would be too difficult to re-identify individuals from it. | No harm or risk | I cannot see any situation where there would be risks or negative consequences. The whole scenario looks positive to me. |
|  |  | Nature of information | the information would be of no value to anyone else (Person 1) Only probabilities will be discovered (Person 2) |
| **Safeguards** | Potential risks are mitigated by a number of safeguards such as trust in the professionals, regulation, and safe protocols. De-identified data, the perception of confidentiality and ensuring only a specific use were also viewed as reasons for the perception of limited or no risk. | Trust in professionals | These doctors are professionals (Person 1) The companies would surely be fully trusted to preserve the information like gold, and only use or share with whom they completely trust (Person 2) |
|  |  | Regulation | it is all very well regulated and tested (Person 1) eventually legislation/ethics will sort those problems out (Person 2) |
|  |  | Systems/protocols | they have good measures in place to prevent them (Person 1) the fact that the information is on a cloud-based system where results/info cannot be downloaded eliminates the risk of information being stolen (Person 2) |
|  |  | De-identified | there is no harm to oneself since the samples have already stripped off my personal identifier (Person 1) My personal information would not be released (Person 2) |
|  |  | Confidential | Generally Health Professionals are far too busy to be bothered gossiping simply for the sake of gossiping. They are required to keep confidential information just that but if they Need to share that information with other Health Professionals to effect a cure then so be it. |
|  |  | Specific use | if they keep their promise to use my genetic info only for specific research study this problem will not arise |
| **There are risks** | Despite indicating there were no perceived risks, some respondents mentioned possible risks either generally or specifically. Risks to privacy and future misuse were mentioned, along with general risks in relation to hacking, police investigations and data errors. Many comments mentioned or implied that these risks were minimal or unlikely to occur. | General | The hacking of cloud information (Person 1) unless the child is a future criminal and a leak identifies them...unlikely (person 2). As the data is anonymous in the shared environment the only perceivable risks would be in any errors that occurred through the analysis process or in recording the resultant data. (person 3) |
|  |  | Misuse | Unless they make a specific bio weapon to kill my genes what else could go wrong?? |
|  |  | Privacy | I don't see anything harmful coming out of this. The only risk is personal identification. But I don't see this as being detrimental to the research. |
| **Benefits** | This theme reflects a general belief that what matters is the pursuit of benefits (whether generally or individually) and that risks are not considered. A more specific subset of comments explicitly mentions that the significance of benefits outweighs the importance of any risks. | General | If sharing helped the child, I can't see any risks at all (Person 1) I can't think of any if the genomes etc are used for research in curing medical conditions (Person 2) |
|  |  | Outweighs risk | I believe that the benefits would far outweigh the negative risks (Person 1) I just feel like even if there were, if it helped my child or other people, it would negate the negative consequences (Person 2) |
|  |  | Personal | the outcomes seem to be to my benefit or potential benefit |

**Question 4: Finally we are interested in what you think is needed to make people feel more comfortable about any potential risks associated with sharing genomic information. Please describe the most important things that could be done in this situation to make you feel more comfortable about sharing if you were the patient/parent/research participant**

| **Theme** | **Definition** | **Subtheme** | **Example quote(s)** |
| --- | --- | --- | --- |
| **Nothing** | Respondents indicated generally that nothing could be done to make them more comfortable about sharing data. Most comments indicated that this was because they were happy doing so, though a minority indicated that nothing would convince them to donate and some simply indicated 'nothing' |  | The strategy laid out seems to have already covered it. I can't think of anything in addition (Person 1) I honestly won't be comfortable since the tech is moving at a much faster pace than the legislation can keep up (Person 2) |
| **Benefits** | This theme reflected that comfort would be obtained just by knowing that benefits for all, science or in general would result from the data sharing in the scenario. The prospects of personal benefits were also mentioned to feel more comfortable. | General | Your DNA is being used for the good benefit of humanity. It will help improve everyone's quality of life and may solve diseases that have lingered for years (Person 1). Just knowing that my genomic information could lead to medical breakthroughs for the benefit of future generations would be comforting enough for me (Person 2). |
|  |  | Personal | if there are any benefits for me in health (Person 1). My family would have information that could benefit them ( Person 2). |
| **Transparency/communication** | Respondents indicated that comfort would be obtained from the provision of increased communication, information and/or transparency in general and specifically associated with the potential benefits and risks of sharing and the procedures (including naming who will have access)related to how and to whom data would be shared. | General | don't hide anything, people can tell when some information is being held back especially medical info |
|  |  | Benefits | Have people understand that sharing genomic information could be helpful for someone in their family or close friends further down the line (Person 1) The knowledge that the information is being used for the right reason would go a long way to keeping people’s minds at ease that the information is being used correctly (Person 2). First an education of the public to inform them of the potential risks - but also the benefits. Consider the activation of certain (negative genes) with environmental factors (Person 3) |
|  |  | Risks | I think being transparent about what the potential risk are that are involve at the beginning stage of the trial as this can help participant be more aware and inform before they fully commit to sharing (Person 1) I think it is most important for transparency at all stages, including information on the risks (including likelihood and consequences) (Person 2) |
|  |  | Procedures | to explain in simple layman language how that information is going to be used and who is going to use them (Person 1) I would like to be informed how the information is being used and by whom (Person 2) |
| **Governance** | Respondents indicated that the presence of law, regulations, protocols or governance procedures in general and specifically related to certain aspects of sharing would make them more comfortable. Some respondents indicated specifically that they would require the government or particular organisations to control data. |  | a contract could be drawn up that all researchers and volunteers have to sign that indicates the protection of the genomic information (Person 1) I think there should be some legal documents that assured the safety of the patient (Person 2) only go to accredited and strictly controlled facilities (Person 3). A Law that gives certain rights to the donor or source of the data or samples (Person 4). The cloud must be run by government and private to ensure security (Person 5). Share information only on trustworthy site such as government site (Person 6) |
| **Trust** | Increasing or obtaining trust in the procedures, the professionals or in general would be required to make people more comfortable to share their data |  | if we want the advantages of this process we must trust that it is used wisely (Person 1) trusting anyone with this genomic information is really risky business, but at the end of the day we have to start to learn to trust, so advancement can be made in the medical world (Person 2) I'd have to be satisfied that I trusted the researcher /organisation to do the right thing (Person 3) |
| **Future use** | Sharing would be dependent upon future use being ethical and information not being misused. Sharing would be dependent only if acceptable third parties are allowed access. |  | If the information is used in an ethical way (Person 1) that it wouldn't/couldn't be used to put individuals in 'boxes' with predictable life outcomes (Person 2). information can only be shared with people who are professionally involved in helping to find a cure for the patient and that the information shall not be shared anywhere else (Person 3). |
| **Commercialisation** | Comfort in sharing would be increased if commercial activity is restricted or that compensation and property rights were made available. | General | Safe guards to ensure it wasn’t available for purchase / obtained by companies who may discriminate (Person 1). it being run by a company in the public sector (Person 2) |
|  |  | IP | any company that stand to make a profit will share the profits with the individuals who contributed to the solution. It is only fair (Person 1). A Law that gives certain rights to the donor or source of the data or samples (Person 2). any company that stand to make a profit will share the profits with the individuals who contributed to the solution. It is only fair (Person 3) |
| **Consent** | Comfort in sharing would increase alongside informed consent. |  | control over how my genetic information is used by third parties by specific opt in (Person 1) Of course, informed consent every step of the way is also important and if anything happens along the way that is unforeseen I would expect to be kept "in the loop" (Person 2). There should be consent given and customers should be allowed to opt out if they don't want to be part of this (Person 3). |
| **Privacy** | Sharing would be conditional of the data not being identified. | Anonymity | That my personal, family and medical information is not known or shared with the research (Person 1) Knowing that this data cannot be linked back to me (Person 2) |
|  | Sharing would be conditional on the information being kept confidential. | Confidentiality | I believe that confidentiality between the parties that are sharing the data is paramount (Person 1) the assurance that the information will remain discreet (Person 2) |
| **Return results** | Respondents reported a desire to receive results, whether they be research results or individual genetic information. |  | Maybe recent findings or a history of results (Person 1) I WOULD LIKE TO GETA COPY IF POSSIBLE SO I COULD LOOK AT MY OWN DNA (Person 2) Complete report to me of my data (Person 3) |
| **Data security** | Comfort would be increased by the presence of data security features or the destruction of samples or data if requested or after use. | General | Make sure information is confidential and have a security feature (Person 1) More security on masking individual’s identities is key (Person 2) |
|  |  | Destroy sample/results | samples destroyed after initial analysis (Person 1) a guarantee that it will be destroyed if requested (Person 2) |
| **Risks are inevitable** | These comments were similar to Nothing, but specifically mentioned that the risks are inevitable and that not much can be done to make one more comfortable about sharing genomic information. |  | I don't think there could be a 100% safety net on this. I guess checks and balances would have to be used but these have been known not to be efficient. Putting things on computers that can be hacked also is not safe (Person 1) Sadly, the world has become a place of dishonesty from paupers to the other end of the spectrum, so trusting anyone with this genomic information is really risky business (Person 2) |
