# Supplementary material for: Context matters in genomic data sharing: a qualitative investigation into responses from the Australian public
Source: BMC Med Genomics. 2023 Apr 1;15(Suppl 3):275. doi: 10.1186/s12920-023-01452-8 (PMC10068139; doi:10.1186/s12920-023-01452-8)
Supplement: Supplementary file 5 — Additional file 5. Title: Participant characteristics. Description: Full breakdown of participant characteristics as (n) and (%) within the whole sample. [file 12920_2023_1452_MOESM5_ESM.docx]

Additional File 5. Participant characteristics.

| Participant characteristic | Female  % within gender category (n) | | Male  % within gender category (n) | |
| --- | --- | --- | --- | --- |
|  | Survey | Population  (Unit 100k) | Survey | Population  (Unit 100k) |
| AGE | | | | |
| Under 50 years of age | 51.26 (61) | 55.37 (51.6) | 41.46 (51) | 57.24 (50.8) |
| % of total | 25.21 | 28.37 | 21.07 | 27.91 |
| 50 years of age or over | 48.74 (58) | 44.63 (41.6) | 58.54 (72) | 42.76 (37.9) |
| % of total | 23.97 | 22.86 | 29.75 | 20.86 |
| EDUCATION | | | | |
| Tertiary | 28.57 (34) | 25.02 (23.3) | 31.71 (39) | 20.84 (18.5) |
| % of total | 14.05 | 12.82 | 16.12 | 10.17 |
| Less than tertiary | 71.43 (85) | 74.98 (69.9) | 68.29 (84) | 79.16 (70.2) |
| % of total | 35.12 | 38.41 | 34.71 | 38.61 |
| PERSONAL DIAGNOSIS OF SERIOUS HEALTH CONDITION | | | | |
| Yes | 19.33 (23) | - | 32.52 (40) | - |
| % of total | 9.50 | - | 16.53 | - |
| No | 75.63 (90) | - | 65.04 (80) | - |
| % of total | 37.19 | - | 33.06 | - |
| I don’t know | 5.04 (6) | - | 2.44 (3) | - |
| % of total | 2.48 | - | 1.24 | - |
| DIAGNOSIS OF SERIOUS HEALTH CONDITION FOR FAMILY MEMBER | | | | |
| Yes | 39.50 (47) | - | 40.65 (50) | - |
| % of total | 19.42 | - | 20.66 | - |
| No | 55.46 (66) | - | 52.85 (65) | - |
| % of total | 27.27 | - | 26.86 | - |
| I don’t know | 5.04 (6) | - | 6.50 (8) | - |
| % of total | 2.48 | - | 3.31 | - |
| ABORIGINAL OR TORRES STRAIT ISLANDER | | | | |
| Yes | 3.36 (4) | 2.14 (1.99) | 1.63 (2) | 2.12 (1.87) |
| % of total | 1.65 | 1.10 | 0.83 | 1.03 |
| No | 96.64 (115) | 97.86 (91.2) | 98.37 (121) | 97.88 (86.8) |
| % of total | 47.52 | 50.13 | 50.00 | 47.74 |
| BACKGROUND | | | | |
| Australian | 71.43 (85) | - | 84.55 (104) | - |
| % of total | 35.12 | - | 42.98 | - |
| Another background | 28.57 (34) | - | 15.45 (19) | - |
| % of total | 14.05 | - | 7.85 | - |
| AUSTRALIAN BORN | | | | |
| Yes | 70.59 (84) | 68.58 (63.9) | 81.30 (100) | 69.05 (61.2) |
| % of total | 34.71 | 35.12 | 41.32 | 33.68 |
| No | 29.41 (35) | 31.42 (29.2) | 18.70 (23) | 30.95 (27.5) |
| % of total | 14.46 | 16.10 | 9.50 | 15.10 |
| URBAN / RURAL |  | |  | |
| Urban | 78.2 (93) | 86.6 (80.7) | 85.4 (105) | 85.6 (75.9) |
| % of total | 38.4 | 44.4 | 43.4 | 41.7 |
| Rural and remote | 21.8 (26) | 13.4 (12.5) | 14.6 (18) | 14.4 (12.8) |
| % of total | 10.7 | 6.9 | 7.4 | 7.0 |
| HAVE CHILDREN |  | |  | |
| Yes | 58.8 (70) | 69.98 (60.1) | 64.2 (79) | - |
| % of total | 28.9 | - | 32.6 | - |
| No | 41.2 (49) | 30.02 (25.8) | 35.8 (44) | - |
| % of total | 20.2 | - | 18.2 | - |

Note: Population data from Australian Bureau of Statistics Census 2016 TableBuilder
